# How to Best Choose the Outer Coarse Mesh in the Domain Decomposition Method of Bank and Jimack

**DOI:** 10.1007/s10013-022-00571-6

**Published:** 2022-05-24

**Authors:** Gabriele Ciaramella, Martin J. Gander, Parisa Mamooler

**Affiliations:** 1 Politecnico di Milano, Dipartimento di Matematica, MOX Lab, Milano, Italy; 2 Université de Genève, Section de Mathématiques, Genève, Switzerland

**Keywords:** Optimized Schwarz method, Bank–Jimack method, Domain decomposition methods, Poisson equation, 65F10, 65N22, 65N55

## Abstract

In Ciaramella et al. (2020) we defined a new partition of unity for the Bank–Jimack domain decomposition method in 1D and proved that with the new partition of unity, the Bank–Jimack method is an optimal Schwarz method in 1D and thus converges in two iterations for two subdomains: it becomes a direct solver, and this independently of the outer coarse mesh one uses! In this paper, we show that the Bank–Jimack method in 2D is an optimized Schwarz method and its convergence behavior depends on the structure of the outer coarse mesh each subdomain is using. For an equally spaced coarse mesh its convergence behavior is not as good as the convergence behavior of optimized Schwarz. However, if a stretched coarse mesh is used, then the Bank–Jimack method becomes faster then optimized Schwarz with Robin or Ventcell transmission conditions. Our analysis leads to a conjecture stating that the convergence factor of the Bank–Jimack method with overlap *L* and *m* geometrically stretched outer coarse mesh cells is $1-O(L^{\frac {1}{2 m}})$.

## Introduction

In 2001, Randolph E. Bank and Peter K. Jimack [3] introduced a new domain decomposition solver for the Bank–Holst adaptive meshing paradigm [2] for the adaptive solution of elliptic partial differential equations. The novel feature of the Bank–Jimack method (BJM) is that each of the subproblems is defined over the entire domain, but outside of the subdomain, a coarse mesh is used. A variant of this new domain decomposition solver using an augmented Lagrange multiplier technique is analyzed in [5] in the context of the abstract Schwarz framework. The BJM in [3] is formulated as a residual correction method, and it is not easy to interpret how and what information is transmitted between subdomains through the outer coarse mesh each subdomain has. A similar difficulty of interpretation existed as well for Additive Schwarz and Restricted Additive Schwarz [11, 15, 25]. This is very different compared to classical domain decomposition methods where this is well understood: classical Schwarz methods [24] exchange information through Dirichlet transmission conditions and use overlap, FETI [12, 13] and Neumann–Neumann methods [6, 22, 23] use Dirichlet and Neumann conditions without overlap, and optimized Schwarz methods (OSMs), which go back to Lions, [20] use Robin or higher order transmission conditions and work with or without overlap, see [14, 15] for an introduction and historic perspective of OSMs. In [10], we showed for a one-dimensional Poisson problem and two subdomains that if one introduces a more general partition of unity, then the BJM becomes an optimal Schwarz method, i.e. a direct solver for the problem converging in two iterations, and this independently of how coarse the outer mesh is. The BJM thus faithfully constructs a Robin type transmission condition involving the Dirichlet to Neumann map in 1D. We analyze here the BJM for the Poisson equation in 2 dimension and two subdomains, and show that with the modified partition of unity, the method can be interpreted as an OSM. Its convergence now depends on the structure of the outer coarse mesh each subdomain uses. In case of equally spaced coarse meshes, we prove that the asymptotic convergence factor is not as good as for an OSM. If one uses however a stretched coarse mesh, i.e. a mesh which becomes gradually more and more coarse in a specific way as one gets further away from the subdomain boundary, the method converges faster than the classical zeroth and second-order OSMs. Based on extensive numerical and asymptotic studies of the analytical convergence factor and the position of coarse points, we conjecture an asymptotic formula for the contraction factor of the BJM. Our analysis also indicates a close relation of the BJM to the class of sweeping type preconditioners [19], since the outer coarse mesh can be interpreted as an implementation of a PML transmission condition, but the BJM is not restricted to sequential decompositions without cross points.

Our paper is organized as follows: in Section 2, the BJM is recalled for a general PDE problem and its generalization by a partition of unity function is introduced (for the influence of partitions of unity on overlapping domain decomposition methods, see [16]). Moreover, for the Laplace problem in two dimensions the BJM is described in detail. The convergence analysis of the BJM is carried out in Section 3, where it is proved to be equivalent to an OSM. This important relation allows us to obtain sharp convergence results. Section 4 is devoted to extensive numerical experiments leading to our conjecture. Finally, our conclusions are presented in Section 5.

## The Bank–Jimack Domain Decomposition Method

In this section, we give a precise description of the BJM, and introduce our model problem and the Fourier techniques that we will use.

### Description of the Method

Let us consider a general self-adjoint^1^ linear elliptic PDE ${\mathscr{L}}u=f$ in a domain *Ω* with homogeneous Dirichlet boundary conditions on *∂Ω*. Discretizing the problem on a global fine mesh leads to a linear system *A****u*** = ***f***, where the matrix *A* is the discrete counterpart of ${\mathscr{L}}$, ***u*** is the vector of unknown nodal values on the global fine mesh, and ***f*** is the load vector.

To describe the BJM, we decompose *Ω* into two overlapping subdomains, *Ω* = *Ω*_1_ ∪*Ω*_2_. The unknown vector ***u*** is partitioned accordingly as $\boldsymbol {u}=\begin {bmatrix} \boldsymbol {u}_{1}^{\top }, \boldsymbol {u}_{s}^{\top }, \boldsymbol {u}_{2}^{\top } \end {bmatrix}^{\top }$, where ***u***_1_ is the vector of unknowns on the nodes in *Ω*_1_ ∖*Ω*_2_, ***u***_*s*_ is the vector of unknowns on the nodes in the overlap *Ω*_1_ ∩*Ω*_2_, and ***u***_2_ is the vector of unknowns on the nodes in *Ω*_2_ ∖*Ω*_1_. We can then write the linear system *A****u*** = ***f*** in block-matrix form,

2.1 $$ \begin{bmatrix} A_{1} & B_{1} & 0\\ B_{1}^{\top} & A_{s} & B_{2}^{\top}\\ 0 & B_{2} & A_{2} \end{bmatrix} \begin{bmatrix} \boldsymbol{u}_{1}\\ \boldsymbol{u}_{s}\\ \boldsymbol{u}_{2} \end{bmatrix} = \begin{bmatrix} \boldsymbol{f}_{1}\\ \boldsymbol{f}_{s}\\ \boldsymbol{f}_{2} \end{bmatrix}. $$

The idea of the BJM is to consider two further meshes on *Ω*, one identical to the original fine mesh in *Ω*_1_, but coarse on *Ω* ∖*Ω*_1_, and one identical to the original fine mesh in *Ω*_2_, but coarse on *Ω* ∖*Ω*_2_. This leads to the two further linear systems

$$ A_{{\varOmega}_{1}} {\boldsymbol v} = T_{2} {\boldsymbol f} \quad\text{and}\quad A_{{\varOmega}_{2}} {\boldsymbol w} = T_{1} {\boldsymbol f}, $$

with

2.2 $$ \begin{array}{ll} &A_{{\varOmega}_{1}} := \begin{bmatrix} A_{1} & B_{1} & 0\\ B_{1}^{\top} & A_{s} & C_{2}\\ 0 & \widetilde{B}_{2} & \widetilde{A}_{2} \end{bmatrix}, \quad \boldsymbol{v} := \begin{bmatrix} \boldsymbol{v}_{1}\\ \boldsymbol{v}_{s}\\ \boldsymbol{v}_{2} \end{bmatrix}, \quad T_{2} := \begin{bmatrix} I_{1} \\ & M_{2} \\ \end{bmatrix}, \\ &A_{{\varOmega}_{2}} := \begin{bmatrix} \widetilde{A}_{1} & \widetilde{B}_{1} & 0\\ C_{1} & A_{s} & B_{2}^{\top}\\ 0 & B_{2} & A_{2} \end{bmatrix}, \quad \boldsymbol{w} := \begin{bmatrix} \boldsymbol{w}_{1}\\ \boldsymbol{w}_{s}\\ \boldsymbol{w}_{2} \end{bmatrix}, \quad T_{1} := \begin{bmatrix} M_{1} \\ & I_{2}\\ \end{bmatrix}, \end{array} $$

where we introduced the restriction matrices *M*_*j*_, *j* = 1,2, to map the fine-mesh vectors ***f***_*j*_ to the corresponding coarse meshes, and *I*_1_ and *I*_2_ are identities of appropriate sizes. Notice that, depending on the chosen discretization scheme, one could get $C_{1} = \widetilde {B}_{1}^{\top }$ and $C_{2} = \widetilde {B}_{2}^{\top }$, which leads to symmetric matrices $A_{{\varOmega }_{1}}$ and $A_{{\varOmega }_{2}}$. However, this symmetry is not generally guaranteed, as we are going to see in the next sections. The BJM as a stationary iteration is then described by Algorithm 1. 
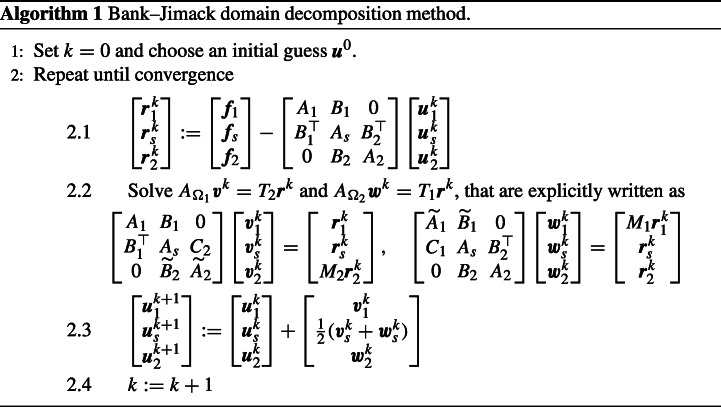


In [10] we studied the BJM for a one-dimensional problem and showed that, in general, it does not lead to a convergent stationary iteration.^2^ To correct this behavior we introduced a discrete partition of unity *D*_1_ + *D*_2_ = *I*, where *I* is the identity matrix and *D*_1_ and *D*_2_ are two matrices that for a one-dimensional problem must have the form (× denote arbitrary entries satisfying the sum condition)

2.3 $$ D_{1}=\text{diag}(1,\times,\dots,\times,0) \quad \text{and} \quad D_{2}=\text{diag}(0,\times,\dots,\times,1). $$

Using these matrices, we modified the BJM by replacing Step 2.3 in Algorithm 1 with

2.4 $$ \begin{bmatrix} \boldsymbol{u}_{1}^{k+1}\\ \boldsymbol{u}_{s}^{k+1}\\ \boldsymbol{u}_{2}^{k+1} \end{bmatrix} := \begin{bmatrix} \boldsymbol{u}_{1}^{k}\\ \boldsymbol{u}_{s}^{k}\\ \boldsymbol{u}_{2}^{k} \end{bmatrix} + \begin{bmatrix} \boldsymbol{v}_{1}^{k}\\ D_{1} \boldsymbol{v}_{s}^{k}+D_{2} \boldsymbol{w}_{s}^{k}\\ \boldsymbol{w}_{2}^{k} \end{bmatrix}. $$

This leads to an iterative method that we proved to be convergent and equivalent to an optimal Schwarz method [18] for the one-dimensional Poisson problem [10]. In [10] we also showed, by direct numerical experiments, that this equivalence does not hold for the two-dimensional Poisson problem. Our goal here is to analyze the convergence of the BJM for two-dimensional problems. Notice that, in what follows, we always refer to BJM as the method obtained by using (2.4) in Algorithm 1.

### The BJM for the Poisson Equation in 2D

Let us consider the problem

**Fig. 1:**
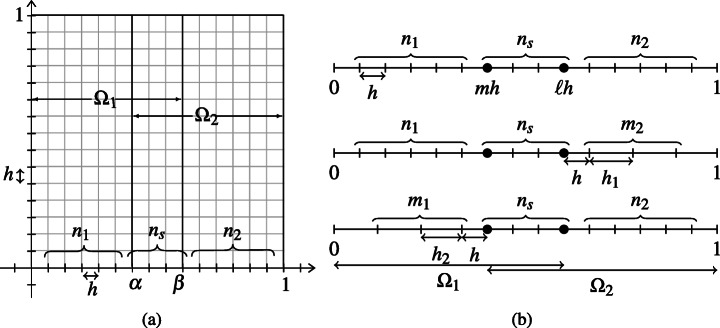
(a) A global fine mesh on *Ω* = (0,1) × (0,1) and the decomposition *Ω* = *Ω*_1_ ∪*Ω*_2_. (b) Global fine mesh in direction *x* (top row), and two partially coarse meshes corresponding to the left subdomain *Ω*_1_ (middle row) and to the right subdomain *Ω*_2_ (bottom row). The black dots represent the *x*-coordinates of the interfaces, namely *mh* and *ℓh*

2.5 $$ \begin{array}{rl} -{\Delta} u=f\quad &\text{in} \quad{\varOmega}=(0,1)\times (0,1),\\ u=0 \quad &\text{on}\quad \partial {\varOmega}, \end{array} $$

where Δ is the Laplace operator and *f* is a sufficiently regular right-hand side function. We consider a uniform grid in *Ω* = (0,1) × (0,1) of *N* interior points in each direction and mesh size $h:=\frac {1}{N+1}$; see, e.g., Fig. 1a. We then discretize (2.5) by a second-order finite-difference scheme, which leads to a linear system *A****u*** = ***f***, where $A \in \mathbb {R}^{N^{2} \times N^{2}}$ is the classical (pentadiagonal) discrete Laplace operator. This system can be easily partitioned as in (2.1). We assume that the vector of unknowns ***u*** is obtained as ${\boldsymbol u}=[{\boldsymbol u}_{1}^{\top },\dots ,{\boldsymbol u}_{N}^{\top }]^{\top }$, where ${\boldsymbol u}_{j} \in \mathbb {R}^{N}$ contains the unknown values on the *j* th column of the grid. In this case, the matrix *A* can be expressed in the Kronecker format *A* = *I*_*y*_ ⊗ *A*_*x*_ + *A*_*y*_ ⊗ *I*_*x*_, where *A*_*x*_ and *A*_*y*_ are *N* × *N* one-dimensional discrete Laplace matrices in directions *x* and *y*, and *I*_*x*_ and *I*_*y*_ are *N* × *N* identity matrices.

The BJM requires two partially-coarse grids. We assume that, in direction *x* our decomposition *Ω* = *Ω*_1_ ∪*Ω*_2_ has *n*_1_ interior points *Ω*_1_ ∖*Ω*_2_, *n*_2_ interior points in *Ω*_2_ ∖*Ω*_1_, and *n*_*s*_ points in *Ω*_1_ ∩*Ω*_2_; see Fig. 1a. For our analysis, the coarsening is performed only in *x*-direction,^3^ as shown in Fig. 1b, while the grid in direction *y* is maintained fine. The partially coarse-grids have *m*_2_ coarse points in *Ω*_2_ ∖*Ω*_1_ (Fig. 1b, middle row) and *m*_1_ coarse points in *Ω*_1_ ∖*Ω*_2_ (Fig. 1b, bottom row) and the corresponding mesh sizes are *h*_1_ and *h*_2_.^4^ If we denote by *A*_*x*,1_ and *A*_*x*,2_ the one-dimensional finite-difference Laplace matrices in *x*-direction, then the partially-coarse matrices $A_{{\varOmega }_{1}}$ and $A_{{\varOmega }_{2}}$ are

2.6 $$ A_{{\varOmega}_{1}} = I_{y} \otimes A_{x,1} + A_{y} \otimes I_{x,1}\quad \text{ and }\quad A_{{\varOmega}_{2}} = I_{y} \otimes A_{x,2} + A_{y} \otimes I_{x,2}, $$

where *I*_*x*,1_ and *I*_*x*,2_ are identities of sizes *n*_1_ + *n*_*s*_ + *m*_2_ and *m*_1_ + *n*_*s*_ + *n*_2_, respectively. Notice that, the matrices *A*_*x*,1_ and *A*_*x*,2_ are classical second-order finite difference matrices in 1D, defined on the union of two uniform grids. Therefore, the only entries that differ from a standard finite-difference formula are the ones corresponding to the stencil across the mesh changes. For example, the five-point formulas for *A*_*x*,1_ on fine and coarse meshes are $(A_{x,1} v)_{j} = \frac {-v_{j-1}+2v_{j} -v_{j+1}}{h^{2}}$, for *j* ≤ *n*_1_ + *n*_2_, and $(A_{x,1} {\boldsymbol v})_{j} = \frac {-v_{j-1}+2v_{j} -v_{j+1}}{{h_{1}^{2}}}$, for *j* ≥ *n*_1_ + *n*_2_ + 2, while at the point across the mesh change (see also Fig. 1), we have

$$ (A_{x,1} v)_{j} = -\frac{2 v_{j-1}}{h(h+h_{1})} + \frac{2 v_{j}}{hh_{1}} -\frac{2 v_{j+1}}{h_{1}(h+h_{1})}\quad\text{ for $j=n_{1}+n_{s}+1$}. $$

The matrices $A_{{\varOmega }_{1}}$ and $A_{{\varOmega }_{2}}$ can be partitioned exactly as in (2.2), and the restriction matrices *T*_1_ and *T*_2_ have now the forms

2.7 $$ T_{1} = I_{y} \otimes \begin{bmatrix} I_{n_{1}+n_{s}} & 0 \\ 0 & \widehat{M}_{2} \\ \end{bmatrix}\quad \text{ and }\quad T_{2} = I_{y} \otimes \begin{bmatrix} \widehat{M}_{1} & 0 \\ 0 & I_{n_{s}+n_{2}} \\ \end{bmatrix}, $$

where $\widehat {M}_{1} \in \mathbb {R}^{m_{1} \times n_{1}}$ and $\widehat {M}_{2} \in \mathbb {R}^{m_{2} \times n_{2}}$ are one-dimensional restriction matrices and $I_{n_{s}+n_{2}}$ and $I_{n_{1}+n_{s}}$ are identity matrices of sizes *n*_*s*_ + *n*_2_ and *n*_1_ + *n*_*s*_. It remains to describe the matrices $D_{1} \in \mathbb {R}^{Nn_{s} \times Nn_{s}}$ and $D_{2} \in \mathbb {R}^{Nn_{s} \times Nn_{s}}$ used in (2.4). These form a partition of unity, that is $D_{1}+D_{2}=I_{Nn_{s}}$, where $I_{Nn_{s}}$ is an identity of size *Nn*_*s*_, and have the forms

2.8 $$ \begin{array}{rl} D_{1} &= I_{y} \otimes \widehat{D}_{1}\quad \text{ with } \widehat{D}_{1} = \text{diag}(1,\times,\dots,\times,0) \in \mathbb{R}^{n_{s}},\\ D_{2} &= I_{y} \otimes \widehat{D}_{2}\quad \text{ with } \widehat{D}_{2} = \text{diag}(0,\times,\dots,\times,1) \in \mathbb{R}^{n_{s}}. \end{array} $$

We have then described all the components that allow us to use the BJM (namely Algorithm 1) for the two-dimensional Poisson problem (2.5).

The choice of discretization by the finite-difference method allows us to perform a detailed convergence analysis based on the diagonalization obtained in Section 2.3.

### A Discrete Fourier Expansion and the *η* −Δ Equation in 1D

The finite-difference matrices *A*, $A_{{\varOmega }_{1}}$ and $A_{{\varOmega }_{2}}$ have similar structures based on Kronecker-product expansions: the matrix components in direction *y* are the same and are not coarsened. Hence, the one-dimensional discrete Laplace matrix *A*_*y*_ appears unchanged in *A*, $A_{{\varOmega }_{1}}$ and $A_{{\varOmega }_{2}}$, while the matrix *A*_*x*_ appearing in *A* is replaced in $A_{{\varOmega }_{1}}$ by *A*_*x*,1_ and in $A_{{\varOmega }_{2}}$ by *A*_*x*,2_.

It is important to notice that *A*_*y*_ is a tridiagonal Toeplitz matrix having values 2/*h*^2^ on the main diagonal and values − 1/*h*^2^ on the first upper and lower diagonals. It is well-known that *A*_*y*_ can be diagonalized as *U*^⊤^*A*_*y*_*U* = Λ, where ${\Lambda }=\text {diag}(\lambda _{1},\dots ,\lambda _{N})$ with *λ*_*j*_ > 0, and the columns of the orthogonal matrix $U\in \mathbb {R}^{N\times N}$ are normalized discrete Fourier sine modes. If one now defines $\widehat {U}:= U \otimes I_{x}$, then it is possible to block-diagonalize *A*,

2.9 $$ \widehat{U}^{\top} A \widehat{U} = I_{y} \otimes A_{x} + {\Lambda} \otimes I_{x} = \begin{bmatrix} A_{x} + \lambda_{1} I_{x} \\ & {\ddots} \\ & & A_{x} + \lambda_{N} I_{x} \\ \end{bmatrix}, $$

where we used the property (*C*_1_ ⊗ *C*_2_)(*C*_3_ ⊗ *C*_4_) = (*C*_1_*C*_3_) ⊗ (*C*_2_*C*_4_), for any matrices *C*_1_, *C*_2_, *C*_3_, and *C*_4_ such that the matrix products *C*_1_*C*_3_ and *C*_2_*C*_4_ can be formed. Defining the vectors $\widehat {{\boldsymbol u}} := \widehat {U}^{\top } {\boldsymbol u}$ and $\widehat {{\boldsymbol f}} := \widehat {U}^{\top } {\boldsymbol f}$ and decomposing them as $\widehat {{\boldsymbol u}} = [ \widehat {{\boldsymbol u}}_{1}^{\top },\dots ,\widehat {{\boldsymbol u}}_{N}^{\top }]^{\top }$ and $\widehat {{\boldsymbol f}} = [\widehat {{\boldsymbol f}}_{1}^{\top } ,\dots ,\widehat {{\boldsymbol f}}_{N}^{\top } ]^{\top }$, we obtain that the linear system *A****u*** = ***f*** can be equivalently written as

2.10 $$ (A_{x} + \lambda_{j} I_{x}) \widehat{{\boldsymbol u}}_{j} = \widehat{{\boldsymbol f}}_{j} \quad\text{ for } j=1,\dots,N. $$

This is the discrete version of a Fourier sine diagonalization of the continuous problem (2.5); see, e.g, [8]. Notice that each component $\widehat {{\boldsymbol u}}_{j} \in \mathbb {R}^{N}$ still represents a vector of nodal values on the *j* th row of the discretization grid. Hence, we can decompose it as $\widehat {{\boldsymbol u}}_{j} = [\widehat {{\boldsymbol u}}_{j,1}^{\top }, \widehat {{\boldsymbol u}}_{j,s}^{\top }, \widehat {{\boldsymbol u}}_{j,2}^{\top }]^{\top }$, where $\widehat {{\boldsymbol u}}_{j,1} \in \mathbb {R}^{n_{1}}$ has values on the nodes in *Ω*_1_ ∖*Ω*_2_, $\widehat {{\boldsymbol u}}_{j,2} \in \mathbb {R}^{n_{2}}$ has values on the nodes in *Ω*_2_ ∖*Ω*_1_, and $\widehat {{\boldsymbol u}}_{j,s} \in \mathbb {R}^{n_{s}}$ has values on the nodes in *Ω*_1_ ∩*Ω*_2_.

Now, using the block-diagonalized form (2.9)–(2.10), we will rewrite the BJM algorithm for each component *j* of $\widehat {{\boldsymbol u}}$. Given an approximation ***u***^*k*^ obtained at the *k* th iteration of Algorithm 1, one can compute $\widehat {{\boldsymbol u}}^{k} = U^{\top } {\boldsymbol u}^{k}$ and $\widehat {{\boldsymbol r}}^{k} = U^{\top } {\boldsymbol r}^{k}$ and rewrite Step 2.1 as

2.11 $$ \widehat{{\boldsymbol r}}_{j}^{k} = \widehat{{\boldsymbol f}}_{j} - (A_{x} + \lambda_{j} I_{x}) \widehat{{\boldsymbol u}}_{j}^{k}\quad \text{ for } j=1,\dots,N. $$

Similarly as for the system *A****u*** = ***f***, we can transform the residual subsystems of Step 2.2. To do so, we define $\widehat {U}_{i}:= U \otimes I_{x,i}$, for *i* = 1,2, such that $\widehat {{\boldsymbol v}}^{k} = \widehat {U}_{1}^{\top } {\boldsymbol v}^{k}$ and $\widehat {{\boldsymbol w}}^{k} = \widehat {U}_{2}^{\top } {\boldsymbol w}^{k}$, and write the subsystems $A_{{\varOmega }_{1}} \boldsymbol {v}^{k} = T_{2} \boldsymbol {r}^{k}$ and $A_{{\varOmega }_{2}} \boldsymbol {w}^{k} = T_{1} \boldsymbol {r}^{k}$ as

$$ \widehat{U}_{1}^{\top} A_{{\varOmega}_{1}} \widehat{U}_{1} \widehat{U}_{1}^{\top}{\boldsymbol v}^{k} = \widehat{U}_{1}^{\top} T_{2} \widehat{U} \widehat{U}^{\top} {\boldsymbol r}^{k} \quad\text{and}\quad \widehat{U}_{2}^{\top} A_{{\varOmega}_{2}} \widehat{U}_{2} \widehat{U}_{2}^{\top} {\boldsymbol w}^{k} = \widehat{U}_{2}^{\top} T_{1} \widehat{U} \widehat{U}^{\top} {\boldsymbol r}^{k}, $$

which allows us to obtain

2.12 $$ \widehat{U}_{1}^{\top} A_{{\varOmega}_{1}} \widehat{U}_{1} \widehat{{\boldsymbol v}}^{k} = \widehat{U}_{1}^{\top} T_{2} \widehat{U} \widehat{{\boldsymbol r}}^{k} \quad\text{and}\quad \widehat{U}_{2}^{\top} A_{{\varOmega}_{2}} \widehat{U}_{2} \widehat{{\boldsymbol w}}^{k} = \widehat{U}_{2}^{\top} T_{1} \widehat{U} \widehat{{\boldsymbol r}}^{k}. $$

Now, using the structures of $A_{{\varOmega }_{i}}$ given in (2.6), we obtain

2.13 $$ \widehat{U}_{i}^{\top} A_{{\varOmega}_{i}} \widehat{U}_{i} = I_{y} \otimes A_{x,i} + {\Lambda} \otimes I_{x,i} = \begin{bmatrix} A_{x,i} + \lambda_{1} I_{x,i} \\ & {\ddots} \\ & & A_{x,i} + \lambda_{N} I_{x,i} \\ \end{bmatrix} $$

for *i* = 1,2, and recalling the matrices *T*_*i*_, defined in (2.7), we get

2.14 $$ \widehat{U}_{1}^{\top} T_{1} \widehat{U} = (U^{\top} \otimes I_{x,1}) \left( I_{y} \otimes \begin{bmatrix} I_{n_{1}+n_{s}} & 0 \\ 0 & \widehat{M}_{2} \\ \end{bmatrix}\right) (U \otimes I_{x}) = I_{y} \otimes \begin{bmatrix} I_{n_{1}+n_{s}} & 0 \\ 0 & \widehat{M}_{2} \\ \end{bmatrix} $$

and

2.15 $$ \widehat{U}_{2}^{\top} T_{2} \widehat{U} = (U^{\top} \otimes I_{x,2}) \left( I_{y} \otimes \begin{bmatrix} \widehat{M}_{1} & 0 \\ 0 & I_{n_{2}+n_{s}} \\ \end{bmatrix}\right) (U \otimes I_{x}) = I_{y} \otimes \begin{bmatrix} \widehat{M}_{1} & 0 \\ 0 & I_{n_{2}+n_{s}} \\ \end{bmatrix}. $$

Replacing (2.13), (2.14) and (2.15) into (2.12), we rewrite the residual systems in Step 2.2 as

2.16 $$ \begin{array}{rl} (A_{x,1} + \lambda_{j} I_{x,1}) \widehat{\boldsymbol{v}}_{j}^{k} &= \begin{bmatrix} I_{n_{1}+n_{s}} & 0 \\ 0 & \widehat{M}_{2} \\ \end{bmatrix} \widehat{\boldsymbol{r}}_{j}^{k}, \\ (A_{x,2} + \lambda_{j} I_{x,2}) \widehat{\boldsymbol{w}}_{j}^{k} &= \begin{bmatrix} \widehat{M}_{1} & 0 \\ 0 & I_{n_{2}+n_{s}} \\ \end{bmatrix} \widehat{\boldsymbol{r}}_{j}^{k} \end{array} $$

for $j=1,\dots ,N$. It remains to study (2.4) (with the matrices *D*_*i*_ defined in (2.8)) that represents Step 2.3. This equation can be written in the compact form

2.17 $$ \boldsymbol{u}^{k+1} = \boldsymbol{u}^{k} + (I_{y} \otimes {D_{1}^{e}}) \boldsymbol{v}^{k} + (I_{y} \otimes {D_{2}^{e}}) \boldsymbol{w}^{k}, $$

where ${D_{1}^{e}} \in \mathbb {R}^{N \times (n_{1}+n_{s}+m_{2})}$ and ${D_{2}^{e}} \in \mathbb {R}^{N \times (m_{1}+n_{s}+n_{2})}$ are given by

$$ {D_{1}^{e}} = \begin{bmatrix} I_{n_{1}} \\ & \widehat{D}_{1} \\ & & 0 \\ \end{bmatrix}\quad \text{ and }\quad {D_{2}^{e}} = \begin{bmatrix} 0 \\ & \widehat{D}_{2} \\ & & I_{n_{2}} \\ \end{bmatrix}. $$

Now, using (2.17) we get

$$ \widehat{U}^{\top} \boldsymbol{u}^{k+1} = \widehat{U}^{\top} \boldsymbol{u}^{k} + \widehat{U}^{\top} (I_{y} \otimes {D_{1}^{e}}) \widehat{U}_{1} \widehat{U}_{1}^{\top} \boldsymbol{v}^{k} + \widehat{U}^{\top} (I_{y} \otimes {D_{2}^{e}})\widehat{U}_{2} \widehat{U}_{2}^{\top} \boldsymbol{w}^{k}, $$

and recalling the structures of ${D_{1}^{e}}$ and ${D_{2}^{e}}$, we obtain

2.18 $$ \begin{bmatrix} \widehat{\boldsymbol{u}}_{j,1}^{k+1}\\ \widehat{\boldsymbol{u}}_{j,s}^{k+1}\\ \widehat{\boldsymbol{u}}_{j,2}^{k+1} \end{bmatrix} = \begin{bmatrix} \widehat{\boldsymbol{u}}_{j,1}^{k}\\ \widehat{\boldsymbol{u}}_{j,s}^{k}\\ \widehat{\boldsymbol{u}}_{j,2}^{k} \end{bmatrix} + \begin{bmatrix} \widehat{\boldsymbol{v}}_{j,1}^{k}\\ \widehat{D}_{1} \widehat{\boldsymbol{v}}_{j,s}^{k}+\widehat{D}_{2} \widehat{\boldsymbol{w}}_{j,s}^{k}\\ \widehat{\boldsymbol{w}}_{j,2}^{k} \end{bmatrix}\quad \text{ for }j=1,\dots,N. $$

Equations (2.11), (2.16) and (2.18) represent the BJM for each discrete Fourier component $\widehat {\boldsymbol {u}}_{j}^{k}$. Clearly the iterative process for each component does not depend on the others, and it suffices to study the convergence of each component separately.

A closer inspection of the matrices in (2.11) and (2.16) reveals that the BJM for one component $\widehat {\boldsymbol {u}}_{j}^{k}$ is exactly the BJM for the solution of a discretized one-dimensional *η* −Δ problem of the form

2.19 $$ \begin{array}{rl} \eta_{j} \widehat{u}_{j}-\partial_{xx}\widehat{u}_{j}&=\widehat{f}_{j} \quad \text{ in }(0,1), \\ \widehat{u}_{j}(0)=\widehat{u}_{j}(1)&=0, \end{array} $$

where $\widehat {u}_{j}$ is the *j* th coefficient of the Fourier sine expansion of *u*, $\widehat {f}_{j}$ is the *j* th Fourier coefficient of *f*, and *η*_*j*_ = (*πj*)^2^. Hence, if we would know a continuous representation of the BJM for the solution to (2.19), then we could perform a Fourier convergence analysis similarly as it is often done at the continuous level for other one-level domain decomposition methods; see, e.g., [7, 8, 14]. This is exactly the focus of Section 3, where we will show that the BJM for the one-dimensional *η* −Δ boundary value problem is an OSM. This equivalence will allow us to perform a detailed Fourier convergence analysis of the BJM.

## Convergence Analysis of the BJM

Motivated by the results in Section 2.3, we study now the BJM for the solution of a one-dimensional discrete *η* −Δ problem and prove that this is equivalent to a discrete OSM, see for example [26]. Our analysis will reveal that the BJM produces implicitly some particular Robin parameters, dependent on *η*, in the equivalent OSM. Since the chosen discretization for the OSM is consistent and convergent, one can pass to the limit from the discrete to the continuous level. Therefore, we will obtain that the continuous limit of the BJM is an OSM, where the Robin parameters are the continuous limits of the discrete Robin parameters of the BJM. Once this equivalence interpretation is established, we will study the dependence of the continuous convergence factor of the BJM with respect to *η* (hence the Fourier frequency), to the size of the overlap, to the number of coarse points and their location.

The main steps of the described analysis are organized in four subsections. In Section 3.1 we recall the OSM, derive its convergence factor at the continuous level and then obtain a discretization based on the finite-difference method for non-uniform grids; see, e.g., [27]. In Section 3.2, we show the equivalence between the BJM and the discrete OSM and discuss the BJM convergence factor in the continuous limit. Sections 3.3 and 3.4 focus on the analysis of the BJM convergence factor for uniform and non-uniform coarse grids.

### The OSM for the One-Dimensional *η* −Δ Equation

To recall the OSM for

3.1 $$ \begin{array}{rcl} \eta u-u_{xx}&=&f\quad \text{ in (0,1)}, \\ u(0)=u(1)&=&0, \end{array} $$

we consider an overlapping domain decomposition (0,1) = (0,*β* := *x*_*ℓ*_) ∪ (*α* := *x*_*m*_,1); see Fig. 1b, top row. Given an appropriate initialization pair $({u_{1}^{0}},{u_{2}^{0}})$, the OSM for (3.1) is

3.2 $$ \arraycolsep0.05em \begin{array}{rcllrcll} \eta {u_{1}^{k}}-\partial_{xx}{u_{1}^{k}}&=&f &\text{in $(0,\beta)$},& \eta {u_{2}^{k}}-\partial_{xx}{u_{2}^{k}}&=&f &\text{in $(\alpha,1)$},\\ {u_{1}^{k}}&=&0 &\text{at $x=0$},& {u_{2}^{k}}&=&0 &\text{at $x=1$},\\ \partial_{x} {u_{1}^{k}}+p_{12}{u_{1}^{k}}&=&\partial_{x} u_{2}^{k-1}+p_{12}u_{2}^{k-1} &\text{at $x=\beta$},\quad & \partial_{x} {u_{2}^{k}}-p_{21}{u_{2}^{k}}&=& \partial_{x} u_{1}^{k-1}-p_{21}u_{2}^{k-1}&\text{at $x=\alpha$}, \end{array} $$

for $k=1,2,\dots $, where *p*_12_ and *p*_21_ are two positive parameters that can be optimized to improve the convergence of the iteration; see, e.g., [14]. This optimization process gives the name *Optimized Schwarz Method* to the scheme (3.2). In fact, the convergence factor of the method depends heavily on *p*_12_ and *p*_21_. To compute this convergence factor, we can assume that *f* = 0 (working by linearity on the error equations). The general solution of the first subproblem in (3.2) with *f* = 0 is of the form $u_{1}(x)=A_{1}e^{\sqrt {\eta }x}+B_{1}e^{-\sqrt {\eta }x}$. Using the boundary condition *u*_1_(0) = 0, we find that *A*_1_ = −*B*_1_ and we thus have $u_{1}(x)=2A_{1}\sinh (\sqrt {\eta }x)$. Similarly, $u_{2}(x)=A_{2}e^{\sqrt {\eta }x}+B_{2}e^{-\sqrt {\eta }x}$, and since *u*_2_(1) = 0, we find that $B_{2}=-A_{2} e^{2\sqrt {\eta }}$ and we thus have $u_{2}(x)=2A_{2}e^{\sqrt {\eta }}\sinh (\sqrt {\eta }(x-1))$. Using the Robin transmission condition at *x* = *α* in the second subproblem of (3.2), we find

$$ \begin{array}{@{}rcl@{}} &&{A_{2}^{k}}\left( e^{\sqrt{\eta}}\sqrt{\eta}\cosh\left( \sqrt{\eta}(\alpha-1)\right)-p_{21}e^{\sqrt{\eta}}\sinh\left( \sqrt{\eta}(\alpha-1)\right) \right)\\ &&=A_{1}^{k-1}\left( \sqrt{\eta}\cosh(\sqrt{\eta}\alpha)-p_{21}\sinh(\sqrt{\eta}\alpha) \right), \end{array} $$

which leads to

3.3 $$ {A_{2}^{k}}=\frac{1}{e^{\sqrt{\eta}}}\frac{\sqrt{\eta}\cosh(\sqrt{\eta}\alpha)-p_{21}\sinh(\sqrt{\eta}\alpha)}{\sqrt{\eta}\cosh(\sqrt{\eta}(\alpha-1))-p_{21}\sinh(\sqrt{\eta}(\alpha-1))}A_{1}^{k-1}. $$

Similarly, using the Robin condition at the point *x* = *β* in the first subproblem of (3.2) we find

3.4 $$ {A_{1}^{k}}=\frac{\sqrt{\eta}\cosh(\sqrt{\eta}(\beta-1))+p_{12}\sinh(\sqrt{\eta}(\beta-1))}{\sqrt{\eta}\cosh(\sqrt{\eta}\beta)+p_{12}\sinh(\sqrt{\eta}\beta)}e^{\sqrt{\eta}}A_{2}^{k-1}. $$

Replacing ${A_{1}^{k}}$ from (3.4) at iteration *k* − 1 into (3.3) shows that the convergence factor over a double iteration of the OSM is

3.5 $$ \begin{array}{@{}rcl@{}} \rho(\eta,p_{12},p_{21},\alpha,\beta)&=&\frac{\sqrt{\eta}\cosh(\sqrt{\eta}(1-\beta))-p_{12}\sinh(\sqrt{\eta}(1-\beta))}{\sqrt{\eta}\cosh(\sqrt{\eta}\beta)+p_{12}\sinh(\sqrt{\eta}\beta)}\\ &&\times\frac{\sqrt{\eta}\cosh(\sqrt{\eta}\alpha)-p_{21}\sinh(\sqrt{\eta}\alpha)}{\sqrt{\eta}\cosh(\sqrt{\eta}(1-\alpha))+p_{21}\sinh(\sqrt{\eta}(1-\alpha))}. \end{array} $$

Notice that the convergence factor *ρ* depends on *η*, the two Robin parameters *p*_12_ and *p*_21_, and on the positions of the interfaces *α* and *β* (hence the length of the overlap *L* := *β* − *α*).

To obtain a discrete formulation of the OSM, we consider two uniform grids of size *h* in the subdomains (0,*β*) and (*α*,1) as the ones shown in Fig. 1b, top row. Using the finite-difference method applied to these grids, we discretize the two subproblems in (3.2) and obtain the linear systems

3.6 $$ A_{\text{OSM,1}} \boldsymbol{u}_{1}^{k} = \boldsymbol{f}_{1} + F_{1} \boldsymbol{u}_{2}^{k-1} \quad \text{ and }\quad A_{\text{OSM,2}} \boldsymbol{u}_{2}^{k} = \boldsymbol{f}_{2} + F_{2} \boldsymbol{u}_{1}^{k-1}, $$

where $A_{\text {OSM},j} \in \mathbb {R}^{(n_{j}+n_{s})\times (n_{j}+n_{s})}$ and $\boldsymbol {f}_{j} \in \mathbb {R}^{n_{j}+n_{s}}$, *j* = 1,2, are

$$ \begin{array}{@{}rcl@{}} A_{\text{OSM,1}} &=& \frac{1}{h^{2}} \begin{small} \begin{bmatrix} 2+\eta h^{2} ~&~ -1~ &~ &~ & \\ -1 ~&~ 2+\eta h^{2} ~&~ -1 ~&~ &\\ ~&~{\ddots}   ~&~ {\ddots} ~&~ {\ddots} ~& \\ ~&~ ~&~ -1 ~&~ 2+\eta h^{2} ~&~ -1 \\ & & &~ -1 ~&~ \frac{2+\eta h^{2}}{2}+p_{12}h \end{bmatrix} \end{small}, \quad \boldsymbol{f}_{1} = \begin{bmatrix} f(x_{1})\\ \vdots\\ f(x_{\ell}) \end{bmatrix},\\ A_{\text{OSM,2}} &=& \frac{1}{h^{2}} \begin{small} \begin{bmatrix} \frac{2+\eta h^{2}}{2}+p_{21}h ~&~ -1 ~&~ & & \\ -1 ~&~ 2+\eta h^{2} ~&~ -1 ~&~ &\\ & {\ddots} & {\ddots} & {\ddots} & \\ & &~ -1 ~&~ 2+\eta h^{2} ~&~ -1 \\ & & &~ -1 ~&~ 2+\eta h^{2} \end{bmatrix} \end{small}, \quad \boldsymbol{f}_{2} = \begin{bmatrix} f(x_{m})\\ \vdots\\ f(x_{N}) \end{bmatrix}, \end{array} $$

and the matrices $F_{1} \in \mathbb {R}^{n_{1}+n_{s} \times n_{2}+n_{s}}$ and $F_{2} \in \mathbb {R}^{n_{2}+n_{s} \times n_{1}+n_{s}}$ are such that

$$ F_{1} \boldsymbol{g}= \begin{small} \begin{bmatrix} 0\\ \vdots\\ 0\\ \left( \frac{p_{12}}{h}-\frac{2+\eta h^{2}}{2h^{2}}\right)(\boldsymbol{g})_{n_{s}}+\frac{1}{h^{2}}(\boldsymbol{g})_{n_{s}+1} \end{bmatrix} \end{small}, \quad F_{2} \boldsymbol{h}= \begin{small} \begin{bmatrix} \left( \frac{p_{21}}{h}-\frac{2+\eta h^{2}}{2h^{2}}\right)(\boldsymbol{h})_{m}+\frac{1}{h^{2}}(\boldsymbol{h})_{m-1}\\ 0\\ \vdots\\ 0\\ \end{bmatrix} \end{small} $$

for any $\boldsymbol {g} \in \mathbb {R}^{n_{2}+n_{s}}$ and $\boldsymbol {h} \in \mathbb {R}^{n_{1}+n_{s}}$. Notice that, since *η*,*p*_12_,*p*_21_ > 0 for any *h* > 0 the matrices *A*_OSM,1_ and *A*_OSM,2_ are strictly diagonally dominant, hence invertible. Therefore, the OSM (3.6) is a stationary method whose standard form (see, e.g., [9]) is

3.7 $$ \begin{bmatrix} \boldsymbol{u}_{1}^{k} \\ \boldsymbol{u}_{2}^{k} \\ \end{bmatrix} = M_{\text{OSM}}^{-1} N_{\text{OSM}} \begin{bmatrix} \boldsymbol{u}_{1}^{k-1} \\ \boldsymbol{u}_{2}^{k-1} \\ \end{bmatrix} + M_{\text{OSM}}^{-1} \begin{bmatrix} \boldsymbol{f}_{1} \\ \boldsymbol{f}_{2} \\ \end{bmatrix}, $$

where $M_{\text {OSM}} = \begin {small} \begin {bmatrix} A_{\text {OSM,1}} & 0 \\ 0 & A_{\text {OSM,2}} \\ \end {bmatrix} \end {small}$ and $N_{\text {OSM}} = \begin {small} \begin {bmatrix} 0 & F_{1} \\ F_{2} & 0 \\ \end {bmatrix} \end {small}$. This is sometimes also called an optimized block Jacobi algorithm; see, e.g., [26]. If convergent, this iterative procedure generates a sequence that converges to the solution of the augmented problem

$$ \begin{bmatrix} A_{\text{OSM,1}} & - F_{1} \\ - F_{2} & A_{\text{OSM,2}} \\ \end{bmatrix} \begin{bmatrix} \boldsymbol{u}_{1} \\ \boldsymbol{u}_{2} \\ \end{bmatrix} = \begin{bmatrix} \boldsymbol{f}_{1} \\ \boldsymbol{f}_{2} \\ \end{bmatrix}. $$

In our analysis, another equivalent form of the the discrete OSM (3.7) will play a crucial role. This is the so-called optimized restricted additive Schwarz (ORAS) method, which is defined as

3.8 $$ \widehat{\boldsymbol{u}}^{k+1} = \widehat{\boldsymbol{u}}^{k} + \widetilde{R}_{1}^{\top} A_{\text{OSM,1}}^{-1} R_{1} \widehat{\boldsymbol{r}}^{k} + \widetilde{R}_{2}^{\top} A_{\text{OSM,2}}^{-1} R_{2} \widehat{\boldsymbol{r}}^{k}, $$

where $\widehat {\boldsymbol {r}}^{k} = \boldsymbol {f} - A \widehat {\boldsymbol {u}}^{k}$, $R_{1} \in \mathbb {R}^{(n_{1}+n_{s}) \times N}$, and $R_{2} \in \mathbb {R}^{(n_{2}+n_{s}) \times N}$ are restriction matrices of the form

3.9 $$ R_{1} = \begin{small} \begin{bmatrix} I_{n_{1}} & 0 & 0 \\ 0 & I_{n_{s}} & 0 \\ \end{bmatrix} \end{small}\quad \text{ and }\quad R_{2} = \begin{small} \begin{bmatrix} 0 & I_{n_{s}} & 0 \\ 0 & 0 & I_{n_{2}} \\ \end{bmatrix} \end{small}, $$

while $\widetilde {R}_{1} \in \mathbb {R}^{(n_{1}+n_{s}) \times N}$ and $\widetilde {R}_{2} \in \mathbb {R}^{(n_{2}+n_{s}) \times N}$ are similar restriction matrices, but corresponding to a non-overlapping decomposition satisfying $\widetilde {R}_{1}^{\top } \widetilde {R}_{1} + \widetilde {R}_{2}^{\top } \widetilde {R}_{2} = I_{N}$; see [26] for more details. It is proved in [26] that (3.8) and (3.7) are equivalent for any *R*_1_ and *R*_2_, as the ones considered in this section, that induce a consistent matrix splitting.

### The BJM as an OSM for the One-Dimensional *η* −Δ Equation

Let us first recall the BJM for the one-dimensional problem (3.1) and state explicitly all the matrices that we need for our analysis. We consider the grids shown in Fig. 1b and the finite-difference method for non-uniform grids; see, e.g., [27]. The full problem on the global fine mesh (Fig. 1b, top row) is

3.10 $$ A\boldsymbol{u}=\boldsymbol{f}, $$

where $A \in \mathbb {R}^{N \times N}$ is a tridiagonal symmetric matrix that we decompose as

$$ A=\begin{bmatrix} A_{1} & B_{1} & 0\\ B_{1}^{\top} & A_{s} & B_{2}^{\top}\\ 0 & B_{2} & A_{2} \end{bmatrix}. $$

The matrices $A_{1} \in \mathbb {R}^{n_{1} \times n_{1}}$, $A_{s} \in \mathbb {R}^{n_{s} \times n_{s}}$, and $A_{2} \in \mathbb {R}^{n_{2} \times n_{2}}$, are tridiagonal and have the form

$$ \frac{1}{h^{2}} \begin{small} \begin{bmatrix} 2+\eta h^{2} ~&~ -1 \\ -1 ~&~ 2+\eta h^{2} ~&~ -1 \\ & {\ddots} & {\ddots} & {\ddots} \\ \end{bmatrix} \end{small}, $$

while $B_{1} \in \mathbb {R}^{n_{1} \times n_{s}}$ and $B_{2} \in \mathbb {R}^{n_{2} \times n_{s}}$ are zero except for one corner entry:

$$ B_{1} = \frac{1}{h^{2}} \begin{small} \begin{bmatrix} \vdots& {\vdots} &{\vdots} & \vdots\\ 0& 0& {\cdots} & 0\\ -1& 0 & {\cdots} & 0 \\ \end{bmatrix} \end{small}\quad \text{ and }\quad B_{2} = \frac{1}{h^{2}} \begin{small} \begin{bmatrix} 0& {\cdots} & 0& -1 \\ 0& {\cdots} &0 & 0\\ \vdots& {\vdots} &{\vdots} & \vdots\\ \end{bmatrix} \end{small}. $$

Hence for a given approximation $\boldsymbol {u}^{k}=[(\boldsymbol {u}_{1}^{k})^{\top },(\boldsymbol {u}_{s}^{k})^{\top },(\boldsymbol {u}_{s}^{k})^{\top }]^{\top }$, the residual ***r***^*k*^ is

3.11 $$ \boldsymbol{r}^{k} = \begin{bmatrix} \boldsymbol{r}_{1}^{k} \\ \boldsymbol{r}_{s}^{k} \\ \boldsymbol{r}_{s}^{k} \\ \end{bmatrix} = \begin{bmatrix} \boldsymbol{f}_{1} \\ \boldsymbol{f}_{s} \\ \boldsymbol{f}_{s} \\ \end{bmatrix} - \begin{bmatrix} A_{1} & B_{1} & 0\\ B_{1}^{\top} & A_{s} & B_{2}^{\top}\\ 0 & B_{2} & A_{2} \end{bmatrix} \begin{bmatrix} \boldsymbol{u}_{1}^{k} \\ \boldsymbol{u}_{s}^{k} \\ \boldsymbol{u}_{s}^{k} \\ \end{bmatrix}. $$

The correction problems on the two partially coarse grids (Fig. 1b, middle and bottom rows), are

3.12 $$ A_{{\varOmega}_{1}} \boldsymbol{v}^{k} = T_{2} \boldsymbol{r}^{k}\quad \text{ and } \quad A_{{\varOmega}_{2}} \boldsymbol{w}^{k} = T_{1} \boldsymbol{r}^{k}, $$

where $A_{{\varOmega }_{1}} \in \mathbb {R}^{(n_{1} + n_{s} + m_{2}) \times (n_{1} + n_{s}+ m_{2})}$, $A_{{\varOmega }_{2}}\in \mathbb {R}^{(n_{2} + n_{s} + m_{1}) \times (n_{2} + n_{s} + m_{1})}$, $T_{1} \in \mathbb {R}^{(n_{2} + n_{s} + m_{1}) \times N}$, and $T_{2} \in \mathbb {R}^{(n_{1} + n_{s} + m_{2}) \times N}$ have the forms given in (2.2), with *A*_1_, *A*_*s*_, *A*_2_, *B*_1_, and *B*_2_ as above. The matrices $C_{1} \in \mathbb {R}^{n_{s} \times m_{1}}$ and $C_{2} \in \mathbb {R}^{n_{s} \times m_{2}}$ are

$$ C_{1} = \frac{1}{h^{2}} \begin{small} \begin{bmatrix} 0& {\cdots} & 0& -1 \\ 0& {\cdots} &0 & 0\\ \vdots& {\vdots} &{\vdots} & \vdots\\ \end{bmatrix} \end{small}\quad \text{ and }\quad C_{2} = \frac{1}{h^{2}} \begin{small} \begin{bmatrix} \vdots& {\vdots} &{\vdots} & \vdots\\ 0 & 0 & {\cdots} & 0 \\ -1 & 0 & {\cdots} & 0 \\ \end{bmatrix} \end{small}. $$

The matrices $\widetilde {A}_{1} \in \mathbb {R}^{m_{1} \times m_{1}}$ and $\widetilde {B}_{1} \in \mathbb {R}^{m_{1} \times n_{s}}$ in the BJM method in Algorithm 1 are

$$ \widetilde{A}_{1} = \frac{1}{{h_{2}^{2}}} \begin{small} \begin{bmatrix} 2+\eta {h_{2}^{2}} & -1 \\ -1 & 2+\eta {h_{2}^{2}} & -1 \\ & {\ddots} & {\ddots} & {\ddots} \\ & -1 & 2+\eta {h_{2}^{2}} & -1 \\ & & \frac{-2 h_{2}}{h+h_{2}} & \frac{2 h_{2}}{h}+\eta {h_{2}^{2}} \\ \end{bmatrix} \end{small}\quad \text{and}\quad \widetilde{B}_{1} = \begin{small} \begin{bmatrix} \vdots& {\vdots} &{\vdots} & \vdots\\ 0 & 0 & {\cdots} & 0 \\ \frac{-2}{h(h+h_{2})} & 0 & {\cdots} & 0 \\ \end{bmatrix} \end{small}, $$

while $\widetilde {A}_{2} \in \mathbb {R}^{m_{2} \times m_{2}}$ and $\widetilde {B}_{2} \in \mathbb {R}^{m_{2} \times n_{s}}$ are

$$ \widetilde{A}_{2} = \frac{1}{{h_{1}^{2}}} \begin{small} \begin{bmatrix} \frac{2 h_{1}}{h}+\eta {h_{1}^{2}} & \frac{-2 h_{1}}{h+h_{1}} \\ -1 & 2+\eta {h_{1}^{2}} & -1 \\ & {\ddots} & {\ddots} & {\ddots} \\ & -1 & 2+\eta {h_{1}^{2}} & -1 \\ & & -1 & 2+\eta {h_{1}^{2}} \\ \end{bmatrix} \end{small}\quad \text{and}\quad \widetilde{B}_{2} = \begin{small} \begin{bmatrix} 0& {\cdots} & 0& \frac{-2}{h(h+h_{1})} \\ 0& {\cdots} &0 & 0\\ \vdots& {\vdots} &{\vdots} & \vdots\\ \end{bmatrix} \end{small}. $$

We do not need to specify the restriction matrices *M*_1_ and *M*_2_, because they multiply the residual components ***r***_1_ and ***r***_2_, which are zero as shown in the upcoming Lemma 3.1. The matrices *M*_*j*_ do not play any role in the convergence of the method if our new partition of unity is used. However, if the original partition of unity proposed in [3] is considered, then they contributes to the convergence behavior. Finally, the partition of unity diagonal matrices $D_{1} \in \mathbb {R}^{n_{s} \times n_{s}}$ and $D_{2} \in \mathbb {R}^{n_{s} \times n_{s}}$ have the structures given in (2.3). Notice that, since *η* > 0, the tridiagonal matrices $\widetilde {A}_{{\varOmega }_{1}}$ and $\widetilde {A}_{{\varOmega }_{1}}$ are strictly diagonally dominant for any *h*,*h*_1_,*h*_2_ > 0, hence invertible.

The BJM in Algorithm 1 consists of iteratively computing the residual (3.11), solving the two correction problems (3.12) and then computing the new approximation using (2.4). We are now ready to prove the equivalence between the BJM and the discrete OSM. To do so, we need an important property of the BJM proved in the next lemma.

#### **Lemma 3.1**

The BJM for the solution of (3.10) (and based on (3.11), (3.12), and (2.4) with all the matrices described above) produces for any initial guess ***u***^0^ and arbitrary partitions of unity satisfying (2.3) zero residual components outside the overlap, $\boldsymbol {r}_{1}^{k}=\boldsymbol {r}_{2}^{k}=0$, for *k* = 1,2,…

#### *Proof*

We only sketch the proof here, since the result is proved in detail in [10]. Moreover, we consider only $\boldsymbol {r}_{1}^{k}$, because the proof for $\boldsymbol {r}_{2}^{k}$ is similar. Using equations (3.11) and (2.4), we compute

$$ \begin{array}{@{}rcl@{}} \boldsymbol{r}_{1}^{k} & =& \boldsymbol{f}_{1}-\left( A_{1}\boldsymbol{u}_{1}^{k}+B_{1}\boldsymbol{u}_{s}^{k}\right)\\ &=&\boldsymbol{f}_{1}-A_{1}\left( \boldsymbol{u}_{1}^{k-1}+\boldsymbol{v}_{1}^{k-1}\right)-B_{1}\left( \boldsymbol{u}_{s}^{k-1} +D_{1}\boldsymbol{v}_{s}^{k-1}+D_{2}\boldsymbol{w}_{s}^{k-1}\right) \\ &=&\boldsymbol{r}_{1}^{k-1}-A_{1}\boldsymbol{v}_{1}^{k-1}-B_{1}\left( D_{1}\boldsymbol{v}_{s}^{k-1}+D_{2}\boldsymbol{w}_{s}^{k-1}\right)\\ &=&B_{1}\boldsymbol{v}_{s}^{k}-B_{1}\left( D_{1}\boldsymbol{v}_{s}^{k-1}+D_{2}\boldsymbol{w}_{s}^{k-1}\right), \end{array} $$

since $\boldsymbol {r}_{1}^{k-1}-A_{1}\boldsymbol {v}_{1}^{k-1}=B_{1}\boldsymbol {v}_{s}^{k-1}$ because of (3.12) at *k* − 1. Now using the structures of *B*_1_, *D*_1_ and *D*_2_ we get

$$ B_{1}D_{1}\boldsymbol{v}_{s}^{k-1}=\frac{1}{h^{2}} \begin{small} \begin{bmatrix} \vdots& {\vdots} &{\vdots} & \vdots\\ 0 & 0 & {\cdots} & 0 \\ -1 & 0 & {\cdots} & 0 \\ \end{bmatrix} \begin{bmatrix} 1 & & & &\\ & \times & & & \\ & & {\ddots} & &\\ & & & \times & \\ & & & & 0 \end{bmatrix} \end{small} \begin{bmatrix} (\boldsymbol{v}_{s,1})^{k-1}\\ \vdots\\ (\boldsymbol{v}_{s,n_{s}})^{k-1} \end{bmatrix} =\frac{1}{h^{2}}\begin{bmatrix} 0\\ \vdots\\ 0\\ (\boldsymbol{v}_{s,1})^{k-1} \end{bmatrix}, $$

independently of the middle elements of *D*_1_,^5^ and thus $B_{1}\boldsymbol {v}_{s}^{k-1}-B_{1}D_{1}\boldsymbol {v}_{s}^{k-1}=0$. By a similarly calculation, one can show that $B_{1}D_{2}\boldsymbol {w}_{s}^{k-1}=0$, also independently of the middle elements of *D*_2_, which proves that $\boldsymbol {r}_{1}^{k}=0$ for *k* = 1,2,… □

Since $\widetilde {A}_{1}$ and $\widetilde {A}_{2}$ are invertible, the Schur-complement matrices $A_{s} - C_{2} \widetilde {A}_{2}^{-1} \widetilde {B}_{2}$ (of $A_{{\varOmega }_{1}}$) and $A_{s} - C_{1} \widetilde {A}_{1}^{-1} \widetilde {B}_{1}$ (of $A_{{\varOmega }_{2}}$) are well-defined and we can compute the entries we need for our analysis using the following lemma.^6^

#### **Lemma 3.2**

The first element of the inverse of the *n* × *n* tridiagonal matrix

$$ T=\begin{bmatrix} a_{1} & b_{1} & & \\ -1 & a & -1& \\ & {\ddots} & {\ddots} & {\ddots} \\ & & -1 & a \end{bmatrix} $$

is given by $\mu (n):=(T^{-1})_{1,1} = \frac {{\lambda _{2}^{n}}-{\lambda _{1}^{n}}}{{\lambda _{2}^{n}}(a_{1}+b_{1}\lambda _{1})-{\lambda _{1}^{n}}(a_{1}+b_{1}\lambda _{2})}$, $\lambda _{1,2}:=\frac {a}{2}\pm \sqrt {\frac {a^{2}}{4}-1}$.

#### *Proof*

The first element of the inverse of *T* is the first component *u*_1_ of the solution of the linear system

$$ T\boldsymbol{u}=\begin{bmatrix} a_{1} & b_{1} & & \\ -1 & a & -1& \\ & {\ddots} & {\ddots} & {\ddots} \\ & & -1 & a \end{bmatrix}\begin{bmatrix} u_{1}\\ u_{2}\\ \vdots\\ u_{n} \end{bmatrix}=\begin{bmatrix} 1\\ 0\\ \vdots\\ 0 \end{bmatrix}. $$

The solution satisfies the recurrence relation − *u*_*j*+ 1_ + *au*_*j*_ − *u*_*j*− 1_ = 0, *j* = 2,3,…,*n* − 1, whose general solution is $u_{j}=C_{1}{\lambda _{1}^{j}}+C_{2}{\lambda _{2}^{j}}$ with *λ*_1,2_ the characteristic roots of *λ*^2^ − *aλ* + 1 = 0 given in the statement of the lemma. The two boundary conditions to determine the constants *C*_1,2_ are

$$ \begin{array}{@{}rcl@{}} a_{1}u_{1}+b_{1}u_{2}&=&a_{1}(C_{1}\lambda_{1}+C_{2}\lambda_{2})+b_{1}(C_{1}{\lambda_{1}^{2}}+C_{2}{\lambda_{2}^{2}})=1,\\ -u_{n-1}+au_{n}&=&-(C_{1}\lambda_{1}^{n-1}+C_{2}\lambda_{2}^{n-1})+a(C_{1}{\lambda_{1}^{n}}+C_{2}{\lambda_{2}^{n}})=0. \end{array} $$

Solving this linear system for *C*_1,2_ gives (using that 3 − *i* = 2 if *i* = 1 and 3 − *i* = 1 if *i* = 2)

$$ C_{i}=\frac{a\lambda_{3-i}^{n}-\lambda_{3-i}^{n-1}} {(a_{1}\lambda_{1}+b_{1}{\lambda_{1}^{2}})(a{\lambda_{2}^{n}}-\lambda_{2}^{n-1})+ (a_{1}\lambda_{2}+b_{1}{\lambda_{2}^{2}})(\lambda_{1}^{n-1}-a{\lambda_{1}^{n}})},\quad i=1,2. $$

Inserting these constants into *u*_*j*_ and evaluating at *j* = 1 gives

$$ u_{1}=\frac{\lambda_{2}^{n-2}(a\lambda_{2}-1) -\lambda_{1}^{n-2}(a\lambda_{1}-1)}{\lambda_{2}^{n-2}(a_{1}+b_{1}\lambda_{1})(a\lambda_{2}-1) -\lambda_{1}^{n-2}(a_{1}+b_{1}\lambda_{2})(a\lambda_{1}-1)}, $$

which upon simplification, using the Vieta relations satisfied by the roots, i.e. *λ*_1_*λ*_2_ = 1 and *λ*_1_ + *λ*_2_ = *a*, leads to the result. □

#### **Lemma 3.3**

The matrices $C_{2}\widetilde {A}_{2}^{-1}\widetilde {B}_{2}$ and $C_{1}\widetilde {A}_{1}^{-1}\widetilde {B}_{1}$ are given by

$$ C_{2}\widetilde{A}_{2}^{-1}\widetilde{B}_{2}= \begin{bmatrix} 0 & & & \\ & {\ddots} & & \\ & & 0 & \\ & & & \frac{{h_{1}^{2}}}{h^{2}}\frac{2\mu(m_{2})}{h(h+h_{1})} \end{bmatrix}, \quad C_{1}\widetilde{A}_{1}^{-1}\widetilde{B}_{1} = \begin{bmatrix} \frac{{h_{2}^{2}}}{h^{2}}\frac{2\mu(m_{1})}{h(h+h_{2})} & & & \\ & 0 & & \\ & & \ \ {\ddots} & &\\ & & & 0 \end{bmatrix}, $$

with the function *μ*(*n*) from Lemma 3.2.

#### *Proof*

For the first result, using the sparsity patterns of *C*_2_ and $\widetilde {B}_{2}$, we obtain

$$ C_{2}\widetilde{A}_{2}^{-1}\widetilde{B}_{2} =\begin{bmatrix} & & \\ & & \\ \frac{-1}{h^{2}} & & \\ \end{bmatrix} \widetilde{A}_{2}^{-1} \begin{bmatrix} & & \frac{-2}{h(h+h_{1})}\\ & & \\ & & \\ \end{bmatrix}=\frac{1}{h^{2}}\begin{bmatrix} 0 & & \\ & {\ddots} & &\\ & & \frac{2(\widetilde{A}_{2}^{-1})_{11}}{h(h+h_{1})} \\ \end{bmatrix}, $$

and we thus need to find the first entry of $\widetilde {A}_{2}^{-1}$. Defining $a_{1}:=\frac {2h_{1}}{h}+\eta {h_{1}^{2}}$, $b_{1}:=\frac {-2h_{1}}{h+h_{1}}$, and $a:=2+\eta {h_{1}^{2}}$, and multiplying by ${h_{1}^{2}}$, we obtain precisely a matrix like in Lemma 3.2,

$$ {h_{1}^{2}}\widetilde{A}_{2}= \begin{small} \begin{bmatrix} a_{1} & b_{1} & & \\ -1 & a & -1 & \\ & {\ddots} & {\ddots} & \ddots\\ & & -1 & a & -1\\ & & & -1 & a \end{bmatrix} \end{small}, $$

and therefore $(({h_{1}^{2}}\widetilde {A}_{2})^{-1})_{11}=\mu (m_{2})$, which shows the first claim. For the second one, it suffices to notice that Lemma 3.2 also holds if the matrix is reordered from top left to bottom right, and can thus be used again. □

Notice that the matrices $C_{2}\widetilde {A}_{2}^{-1}\widetilde {B}_{2}$ and $C_{1}\widetilde {A}_{1}^{-1}\widetilde {B}_{1}$ are operators which are non-zero only on the last column of the overlap. Therefore, as we are going to see in the rest of this section, they can be interpreted as generalized Robin boundary conditions. Now, using the Schur-complements $A_{s} - C_{2} \widetilde {A}_{2}^{-1} \widetilde {B}_{2}$ (of $A_{{\varOmega }_{1}}$) and $A_{s} - C_{1} \widetilde {A}_{1}^{-1} \widetilde {B}_{1}$ (of $A_{{\varOmega }_{2}}$), we can introduce the matrices $\widehat {A}_{1}$ and $\widehat {A}_{2}$:

$$ \widehat{A}_{1} := \begin{bmatrix} A_{1} & B_{1} \\ B_{1}^{\top} & A_{s} - C_{2} \widetilde{A}_{2}^{-1} \widetilde{B}_{2} \end{bmatrix}\quad \text{ and }\quad \widehat{A}_{2} := \begin{bmatrix} A_{s} - C_{1} \widetilde{A}_{1}^{-1} \widetilde{B}_{1} & B_{2}^{\top} \\ B_{2} & A_{2} \end{bmatrix}, $$

which allow us to prove the following result.

#### **Lemma 3.4**

The matrices $\widehat {A}_{1}$ and $\widehat {A}_{2}$ are invertible and the inverses of $A_{{\varOmega }_{1}}$ and $A_{{\varOmega }_{2}}$ have the forms

3.13 $$ A_{{\varOmega}_{1}}^{-1} = \begin{small} \begin{bmatrix} \widehat{A}_{1}^{-1} & 0 \\ - \overline{B}_{1} \widehat{A}_{1}^{-1} & I_{m_{2}} \\ \end{bmatrix} \end{small} \begin{small} \begin{bmatrix} I_{n_{1}} & 0 & 0 \\ 0 & I_{n_{s}} & -C_{2} \widetilde{A}_{2}^{-1} \\ 0 & 0 & \widetilde{A}_{2}^{-1} \\ \end{bmatrix} \end{small} $$

and

3.14 $$ A_{{\varOmega}_{2}}^{-1} = \begin{small} \begin{bmatrix} I_{m_{2}} & -\overline{B}_{2} \widehat{A}_{2}^{-1} \\ 0 & \widehat{A}_{2}^{-1} \\ \end{bmatrix} \end{small} \begin{small} \begin{bmatrix} \widetilde{A}_{1}^{-1} & 0 & 0 \\ -C_{2} \widetilde{A}_{2}^{-1} & I_{n_{s}} & 0 \\ 0 & 0 & I_{n_{2}} \\ \end{bmatrix} \end{small}, $$

where $\overline {B}_{1} = [ 0 , \widetilde {A}_{2}^{-1}\widetilde {B}_{2} ]$ and $\overline {B}_{2} = [ \widetilde {A}_{1}^{-1}\widetilde {B}_{1} , 0 ]$.

#### *Proof*

We prove the result for $\widehat {A}_{1}$. The proof for $\widehat {A}_{2}$ can be obtained exactly by the same arguments. Recalling that *η* > 0, a direct inspection of the matrix $A_{{\varOmega }_{1}}$ reveals that it is strictly diagonally dominant. Hence, $\det (A_{{\varOmega }_{1}})\neq 0$. Now, consider the block structure of $A_{{\varOmega }_{1}}$ given in (2.2). Since $\widetilde {A}_{2}$ is invertible, we factorize $A_{{\varOmega }_{1}}$ as

$$ \begin{small} \begin{bmatrix} A_{1} & B_{1} & 0\\ B_{1}^{\top} & A_{s} & C_{2}\\ 0 & \widetilde{B}_{2} & \widetilde{A}_{2} \end{bmatrix} = \begin{bmatrix} I_{n_{1}} & 0 & 0\\ 0 & I_{n_{s}} & C_{2}\\ 0 & 0 & \widetilde{A}_{2}\\ \end{bmatrix} \begin{bmatrix} A_{1} & B_{1} & 0\\ B_{1}^{\top} & A_{s}-C_{2} \widetilde{A}_{2}^{-1} \widetilde{B}_{2} & 0\\ 0 & \widetilde{A}_{2}^{-1} \widetilde{B}_{2} & I_{m_{2}} \\ \end{bmatrix} \end{small}, $$

where $I_{n_{1}}$, $I_{n_{s}}$, and $I_{m_{2}}$ are identity matrices of sizes *n*_1_, *n*_*s*_, and *m*_2_. This factorization allows us to write $0 \neq \det (A_{{\varOmega }_{1}}) = \det (\widetilde {A}_{2})\det (\widehat {A}_{1})$, which implies that $\det (\widehat {A}_{1})\neq 0$. Now, a straightforward calculation using the previous factorization allows us to get (3.13). □

Now, we notice that the BJM can be written (using (3.12) and (2.4)) in the compact form

3.15 $$ \boldsymbol{u}^{k+1} = \boldsymbol{u}^{k} + \widetilde{T}_{1} A_{{\varOmega}_{1}}^{-1} T_{1} \boldsymbol{r}^{k} + \widetilde{T}_{2} A_{{\varOmega}_{2}}^{-1} T_{2} \boldsymbol{r}^{k}, $$

where the block-diagonal matrices $\widetilde {T}_{1} \in \mathbb {R}^{(n_{1}+ n_{s}+m_{2})\times N}$ and $\widetilde {T}_{2} \in \mathbb {R}^{(m_{1}+ n_{s}+n_{2})\times N}$ are

$$ \widetilde{T}_{1} = \begin{small} \begin{bmatrix} I_{n_{1}} & 0 & 0\\ 0 & D_{1} & 0\\ 0 & 0 & 0 \\ \end{bmatrix} \end{small}\quad \text{ and }\quad \widetilde{T}_{2} = \begin{small} \begin{bmatrix} 0 & 0 & 0\\ 0 & D_{2} & 0\\ 0 & 0 & I_{n_{2}} \\ \end{bmatrix} \end{small}. $$

A direct calculation using Lemma 3.1 (hence that $\boldsymbol {r}_{1}^{k}=0$ and $\boldsymbol {r}_{2}^{k}=0$) and Lemma 3.4 (hence the formulas (3.13) and (3.14)) allows us to obtain

$$ \widetilde{T}_{1} A_{{\varOmega}_{1}}^{-1} T_{1} \boldsymbol{r}^{k} = \begin{small} \begin{bmatrix} I_{n_{1}} & 0 \\ 0 & D_{1} \\ 0 & 0 \\ \end{bmatrix} \end{small} \widehat{A}_{1}^{-1} R_{1} \boldsymbol{r}^{k}\quad \text{ and }\quad \widetilde{T}_{2} A_{{\varOmega}_{2}}^{-1} T_{2} \boldsymbol{r}^{k} = \begin{small} \begin{bmatrix} 0 & 0 \\ D_{2} & 0 \\ 0 & I_{n_{2}} \\ \end{bmatrix} \end{small} \widehat{A}_{2}^{-1} R_{2} \boldsymbol{r}^{k}, $$

where the matrices *R*_1_ and *R*_2_ are the ones given in (3.9). Since the results proved in Lemma 3.1 are independent of the middle diagonal entries of *D*_1_ and *D*_2_, we can choose them such that the equalities

3.16 $$ \widetilde{R}_{1}^{\top} = \begin{small} \begin{bmatrix} I_{n_{1}} & 0 \\ 0 & D_{1} \\ 0 & 0 \\ \end{bmatrix} \end{small}\quad \text{ and }\quad \widetilde{R}_{2}^{\top} = \begin{small} \begin{bmatrix} 0 & 0 \\ D_{2} & 0 \\ 0 & I_{n_{2}} \\ \end{bmatrix} \end{small} $$

are fulfilled. Therefore, the BJM (3.15) becomes

3.17 $$ \boldsymbol{u}^{k+1} = \boldsymbol{u}^{k} + \widetilde{R}_{1}^{\top} \widehat{A}_{1}^{-1} R_{1} \boldsymbol{r}^{k} + \widetilde{R}_{2}^{\top} \widehat{A}_{2}^{-1} R_{2} \boldsymbol{r}^{k}, $$

which is already very similar to the ORAS method (3.8). Now, a direct comparison of $\widehat {A}_{1}$ and *A*_OSM,1_, which uses the results of Lemma 3.3, reveals that they are equal except for the bottom-right corner elements, which are

$$ \begin{array}{@{}rcl@{}} (A_{\text{OSM},1})_{n_{1}+n_{s} , n_{1}+n_{s}} &=& \frac{2+\eta h^{2}}{2 h^{2}}+\frac{p_{12}}{h}, \\ (\widehat{A}_{1})_{n_{1}+n_{s} , n_{1}+n_{s}} &=& \frac{2+\eta h^{2}}{h^{2}} - \frac{2{h_{1}^{2}}}{h^{3}(h+h_{1})}\mu(m_{2}). \end{array} $$

Similarly, $\widehat {A}_{2}$ and *A*_OSM,2_ are equal except for the top-left corner elements, which are

$$ \begin{array}{@{}rcl@{}} (A_{\text{OSM},2})_{1,1} &=& \frac{2+\eta h^{2}}{2 h^{2}}+\frac{p_{21}}{h}, \\ (\widehat{A}_{2})_{1,1} &=& \frac{2+\eta h^{2}}{h^{2}} - \frac{2{h_{2}^{2}}}{h^{3}(h+h_{2})}\mu(m_{1}). \end{array} $$

Therefore, if one chooses

3.18 $$ p_{12} := \frac{2+\eta h^{2}}{2 h} - \frac{2{h_{1}^{2}}}{h^{2}(h+h_{1})}\mu(m_{2}) \quad \text{and}\quad p_{21} := \frac{2+\eta h^{2}}{2 h} - \frac{2{h_{2}^{2}}}{h^{2}(h+h_{2})}\mu(m_{1}), $$

then $\widehat {A}_{j} = A_{\text {OSM},j}$ for *j* = 1,2. Replacing this equality into (3.17), we obtain that the BJM is equivalent to the ORAS method (3.8), and hence to the discrete OSM (3.6). We summarize our findings in the following theorem.

#### **Theorem 3.5**

If the partition of unity matrices *D*_1_ and *D*_2_ have the forms (2.3) and are chosen such that the equalities (3.16) hold, and if the Robin parameters of the discrete OSM (3.6) are chosen as in (3.18), then the BJM is equivalent to the ORAS method (3.8) and to the discrete OSM (3.6).

Notice that Theorem 3.5 has the following important consequence. Since the discrete OSM (3.6) is obtained by a consistent and convergent discretization of the continuous OSM (3.2), we find that, in the limit for $h\rightarrow 0$, the continuous counterpart of the BJM is the OSM (3.2). This will allow us to study in Sections 3.3 and 3.4 the convergence factor of the BJM at the continuous level. For this purpose, from now on, we denote by *p*_12_(*h*,*η*,*h*_1_) and *p*_21_(*h*,*η*,*h*_2_) the two Robin parameters of (3.18) to stress their dependence on the discretization size *h*, the (Fourier) parameter *η* and the coarse mesh sizes *h*_1_ and *h*_2_. Notice that *μ*(*m*_2_) and *μ*(*m*_1_) in (3.18) depend on *h*, *h*_1_, *h*_2_ and *η* (see Lemma 3.3). Recalling the results obtained in Section 3.1, the continuous BJM convergence factor is given by (3.5), where *p*_12_ and *p*_21_ are the limits for $h\rightarrow 0$ (with *m*_1_ and *m*_2_ fixed) of the parameters chosen in Theorem 3.5.

It is important to remark at this point that the first coarse points, namely the point *n*_1_ + *n*_*s*_ + 1 for the first mesh and the point *m*_1_ for the second mesh, are located at distance *h* from the interfaces. With this choice we were able to define discrete finite-difference derivatives across these points and in Sections 3.3 and 3.4 we will take limits for $h\rightarrow 0$, while keeping the numbers *m*_1_ and *m*_2_ of the coarse points fixed.

Finally, we wish to remark that all the calculations performed in this section, except for the precise formulas for *μ*(*m*_2_) and *μ*(*m*_1_) in Lemma 3.3, remain valid if, instead of uniform coarse grids, one considers two coarse grids which are non-uniform, in the sense that the *m*_1_ points in *Ω*_1_ ∖*Ω*_2_ and the *m*_2_ points in *Ω*_2_ ∖*Ω*_1_ are not uniformly distributed, leading to invertible matrices $\widetilde {A}_{1}$ and $\widetilde {A}_{2}$. Therefore, the equivalence between BJM and OSM remains valid also in the case of non-uniform coarse grids.

### Uniform Coarse Grid

The goal of this section is to study the contribution of uniform coarse grids to the convergence of the BJM for the solution to (2.5). For simplicity, we assume that the two partially coarse grids have the same number of coarse points *m* := *m*_1_ = *m*_2_. To satisfy this condition, we fix the size of the overlap *L* and choose $\alpha =\frac {1-L}{2}$ and $\beta =\frac {1+L}{2}$. In this case, we also have that $h_{1}=h_{2}=\frac {1-\beta -h}{m}$. We consider the cases of *m* = 2, *m* = 3, and *m* = 4 coarse points.

For the sake of clarity, we first summarize the structure of our analysis. For each given *m* ∈{2,3,4}, we first consider the corresponding BJM Robin parameters, whose explicit formulas can be obtained as in Lemma 3.3, and then pass to the limit for $h \rightarrow 0$ to get their continuous counterparts. These continuous parameters will be replaced into the formula (3.5), which will give us the continuous convergence factor of the BJM corresponding to the given *m*, to a fixed (Fourier) parameter *η*, and to the size of the overlap *L*. For fixed *m* and given values of *L* we will numerically compute the maximum of the convergence factor with respect to the (Fourier) parameter *η*. This will allow us to study the deterioration of the contraction factor for decreasing size *L* of the overlap. While performing this analysis, we compare the convergence of the BJM to the one of the OSM with optimized parameter.

From the convergence factor *ρ* of the OSM in (3.5), we see that choosing

$$ p_{12}^{\ast} = \sqrt{\eta}\coth(\sqrt{\eta}(1-\beta)) \quad \text{and}\quad p_{21}^{*}=\sqrt{\eta}\coth(\sqrt{\eta}\alpha) $$

gives *ρ* = 0 for the frequency *η*. These are thus the optimal parameters for this frequency, and make the OSM a direct solver for the corresponding error component.

For *m* = 2 coarse points, proceeding as in the proof of Lemma 3.2 to compute the corresponding *μ*(*m*_2_) = *μ*(*m*_1_) and using (3.18), we get the (discrete) BJM Robin parameters

3.19 $$ \begin{array}{ll} &\displaystyle{p_{12}=\frac{1}{h}+\frac{\eta h}{2}-h E_{2}(h_{1}) \quad\text{and}\quad p_{21}=\frac{1}{h}+\frac{\eta h}{2}-h E_{2}(h_{2})},\\ & \displaystyle{E_{2}(\tilde{h}):=\frac{2(\eta \tilde{h}^{2}+2)\tilde{h}}{h^{2}(\eta^{2}h^{2}\tilde{h}^{3}+\eta^{2}h\tilde{h}^{4}+2\eta h^{2}\tilde{h}+4\eta h\tilde{h}^{2}+2\eta \tilde{h}^{3}+2h+4\tilde{h})}}. \end{array} $$

Recalling that $h_{1}=h_{2}=\frac {1-\beta -h}{2}$ and taking the limit for $h \rightarrow 0$, we obtain

3.20 $$ \hat{p}_{12}:=\lim_{h \rightarrow 0} p_{12} = R_{2}(1-\beta),~~ \hat{p}_{21}:=\lim_{h \rightarrow 0} p_{21} = R_{2}(\alpha),~~ R_{2}(\tilde{L}):= \frac{\tilde{L}^{4}\eta^{2}+16\tilde{L}^{2}\eta+32}{4\tilde{L}^{3}\eta+32\tilde{L}}. $$

**Fig. 2:**
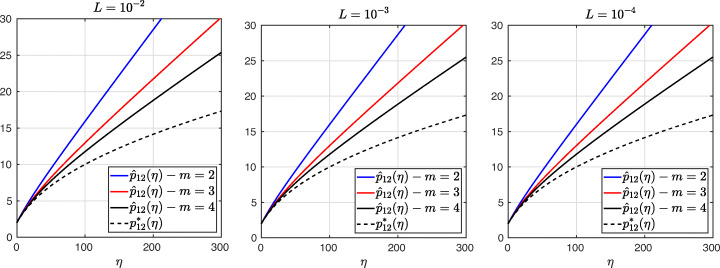
Comparison of the Robin parameters $p_{12}^{\ast }$ of the OSM and $\hat {p}_{12}$ of the BJM for *m* = 2,3,4 (uniformly distributed) coarse points and overlap *L* = 10^− 2^ (left), *L* = 10^− 3^ (middle), *L* = 10^− 4^ (right)

**Fig. 3:**
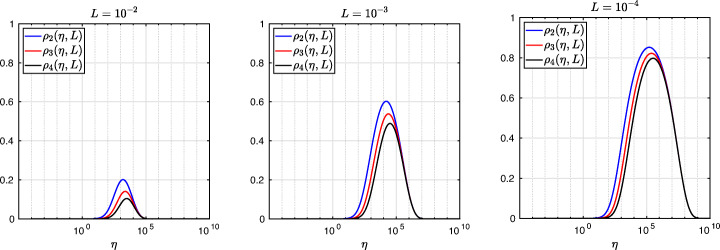
Convergence factors *ρ*_*m*_(*η*,*L*) as functions of *η* and for *m* = 2,3,4 (uniformly distributed) coarse points and *L* = 10^− 2^ (left), *L* = 10^− 3^ (middle), *L* = 10^− 4^ (right)

**Fig. 4:**
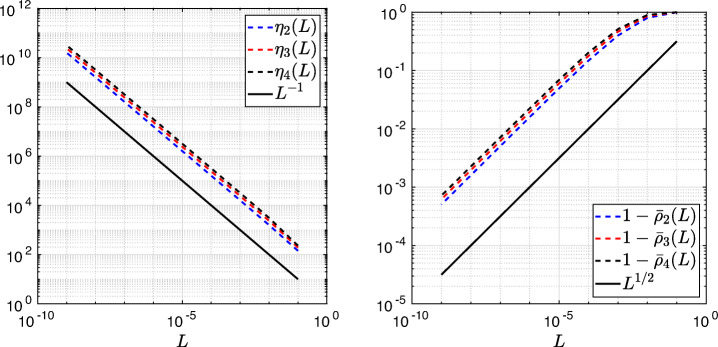
Left: *η*_*m*_(*L*) versus *L* for *m* = 2,3,4. Right: $1-\bar {\rho }_{m}(L)$ versus *L* for *m* = 2,3,4 (uniformly distributed) coarse points

We see that the Robin parameters $\hat {p}_{12}$ and $\hat {p}_{21}$ are rational functions of the Fourier parameter *η* with coefficients depending on the outer subdomain sizes 1 − *β* and *α*. In Fig. 2, we compare the Robin parameter $\hat {p}_{12}$ of the BJM for *m* = 2 (blue line) with the optimal Robin parameter $p_{12}^{\ast }$ of the OSM (black dashed line) for three different values of the overlap *L*. We observe that for small *η* the Robin parameters of both methods are quite close, which indicates that the BJM method performs well for low-frequency error components. This is clearly visible in Fig. 3, where we plot the corresponding convergence factors (as functions of *η*) inserting $\hat {p}_{12}$ and $\hat {p}_{12}$ into (3.5)^7^ for two different overlaps *L*, using $\alpha =\frac {1-L}{2}$ and $\beta =\frac {1+L}{2}$. We also see that the convergence factor clearly has a maximum at some *η*_2_(*L*), whose corresponding error mode converges most slowly, and convergence deteriorates when *L* becomes small. In Fig. 4 (left), we present the value *η*_2_(*L*) as functions of *L* and observe that it grows like *O*(*L*^− 1^). The corresponding contraction factor, namely $\bar {\rho }_{2}(L) := \max \limits _{\eta } \rho _{2}(\eta ,L) := \max \limits _{\eta }\rho \big (\eta ,\hat {p}_{12}(\eta ,L),\hat {p}_{21}(\eta ,L),\alpha =\frac {1-L}{2},\beta =\frac {1+L}{2}\big )$, is shown as function of *L* in Fig. 4 (right-dashed blue line, represented as 1 − *ρ*_2_(*L*)). Here, one can observe clearly that as *L* gets smaller the convergence deteriorates with an order *O*(*L*^1/2^).

Let us now discuss the behavior $\bar {\rho }_{2}(L)=1-O(L^{\frac {1}{2}})$ shown in Fig. 4 (right): it was proved in [14] that the convergence factor of the OSM with overlap *L* behaves like $\rho _{OSM}^{\star }=1- O(L^{\frac {1}{3}})$ with Robin transmission conditions, and $\rho _{OSM}^{\star }=1- O(L^{\frac {1}{5}})$ with second-order (Ventcell) transmission conditions. Hence, the OSM performs better than the BJM with a uniform coarse grid with *m* = 2 uniformly distributed coarse points,^8^ since convergence deteriorates more slowly when the overlap *L* goes to zero.

We have seen that, for only two points the BJM is already a good method for low frequencies, since the parameters $\hat {p}_{12}$ and $\hat {p}_{21}$ are very close to the optimal ones $p_{12}^{\ast }$ and $p_{21}^{\ast }$ for relatively small *η*. However, the convergence factor deteriorates with *L* faster than for the OSM. It is natural to ask: does the behavior of the BJM improve if more coarse points are used? The answer is surprisingly negative! In fact, the convergence factor remains of order $1-O(L^{\frac {1}{2}})$. To see this, we now repeat the analysis for uniform coarse grids with *m* = 3 and *m* = 4 points. For *m* = 3, we find the analog of (3.19) with $E_{2}(\tilde {h})$ replaced by

$$ E_{3}(\tilde{h})= \frac{2(\eta^{2}\tilde{h}^{4}+4\eta \tilde{h}^{2}+3)\tilde{h}}{h^{2}(\eta^{3}h^{2}\tilde{h}^{5}+\eta^{3}h\tilde{h}^{6}+4\eta^{2}h^{2}\tilde{h}^{3}+6\eta^{2}h\tilde{h}^{4}+2\eta^{2}\tilde{h}^{5} +3\eta h^{2}\tilde{h}+9\eta h \tilde{h}^{2}+8\eta \tilde{h}^{3}+2h+6\tilde{h})}, $$

and for the corresponding optimized parameters when *h* goes to zero the analog of (3.20) with the rational function $R_{2}(\tilde {L})$ replaced by

$$ R_{3}(\tilde{L})= \frac{\tilde{L}^{6}\eta^{3}+54\tilde{L}^{4}\eta^{2}+729\tilde{L}^{2}\eta+1458}{6\tilde{L}^{5}\eta^{2}+216\tilde{L}^{3}\eta+1458\tilde{L}}. $$

In Fig. 2 (red lines) we show the Robin parameters of the BJM with *m* = 3 coarse points as a function of *η* and we compare it to the optimal Robin parameters of the OSM. We observe that they are closer compared to the *m* = 2 point case. This seems to suggest an improvement of the convergence factor, but the plots of the convergence factor in Fig. 3 show that this improvement is only minor compared to the case of *m* = 2 coarse mesh points. This is also confirmed by the results in Fig. 4 (right): we see that $\bar {\rho }_{3}=1-O(L^{\frac {1}{2}})$, similar to the *m* = 2 coarse point case. The same happens for the *m* = 4 coarse mesh point case, where

$$ R_{4}(\tilde{L}) = \frac{{\tilde{L}^{8}} {\eta^{4}}+128 {\tilde{L}^{6}} {\eta^{3}}+5120 {\tilde{L}^{4}} {\eta^{2}}+65536 {\tilde{L}^{2}} \eta +131072}{8 {\tilde{L}^{7}} {\eta^{3}}+768 {\tilde{L}^{5}} {\eta^{2}}+20480 {\tilde{L}^{3}} \eta +131072 \tilde{L}} $$

and we show the corresponding contraction factor in Figs. 3 (black lines) and 4 (right). Again we see that $\bar {\rho }_{4}(L) = 1 - O(L^{1/2})$.

We thus conclude that the convergence factor of the BJM with a uniform coarse grid always behaves as $1-O(L^{\frac {1}{2}})$ independently of the number of coarse points of the grids. This shows that the OSM has a better convergence factor compared to the BJM with uniform coarse grids since its convergence factor behaves as $1-O(L^{\frac {1}{3}})$, but BJM with uniform coarse grids converges better than classical Schwarz, which has a convergence factor 1 − *O*(*L*), see [14]. Is the uniformity of the coarse grids the limiting factor for BJM? We address this in the next section.

### Stretched Coarse Grid

**Fig. 5:**
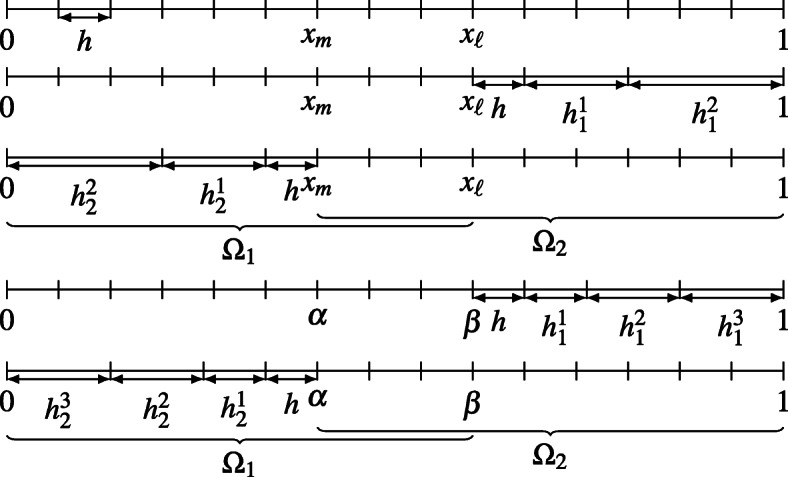
First top row: global uniform grid. Second and third rows: stretched coarse grids with 2 points. Fourth and fifth rows: stretched coarse grids with 3 points

We now consider stretched coarse grids, and start with *m* = 2 non-uniformly distributed coarse points with grid sizes ${h_{1}^{1}}$, ${h_{1}^{2}}$, ${h_{2}^{1}}$, and ${h_{2}^{2}}$, see Fig. 5 (second and third rows). Using the finite-difference method, we discretize our problem and obtain the two linear systems $A_{{\varOmega }_{1}}\boldsymbol {v}=T_{2} \boldsymbol {f}$ and $A_{{\varOmega }_{2}}\boldsymbol {w}=T_{1} \boldsymbol {f}$, where $A_{{\varOmega }_{1}}$ and $A_{{\varOmega }_{2}}$ have the block-structures given in (2.2) with the blocks corresponding to the coarse parts of the grids that are

$$ \begin{array}{@{}rcl@{}} \widetilde{A}_{1}&=& \begin{small} \begin{bmatrix} \frac{2}{{h_{2}^{1}}{h_{2}^{2}}}+\eta & \frac{-2}{{h_{2}^{1}}({h_{2}^{1}}+{h_{2}^{2}})}\\ \frac{-2}{{h_{2}^{1}}(h+{h_{2}^{1}})} & \frac{2}{h {h_{2}^{1}}}+\eta \end{bmatrix} \end{small},\quad \widetilde{B}_{1}= \begin{small} \begin{bmatrix} 0 & 0 \\ \frac{-2}{h(h+{h_{2}^{1}})} & 0 \end{bmatrix} \end{small}, \quad C_{1}= \begin{small} \begingroup \renewcommand*{\arraystretch}{0.95} \begin{bmatrix} 0 & \frac{-1}{h^{2}}\\ 0 & 0 \end{bmatrix} \endgroup \end{small}, \\ \widetilde{A}_{2}&=& \begin{small} \begin{bmatrix} \frac{2}{h{h_{1}^{1}}}+\eta & \frac{-2}{{h_{1}^{1}}(h+{h_{1}^{1}})}\\ \frac{-2}{{h_{1}^{1}}({h_{1}^{1}}+{h_{1}^{2}})} & \frac{2}{{h_{1}^{1}}{h_{1}^{2}}}+\eta \end{bmatrix} \end{small}, \quad \widetilde{B}_{2}= \begin{small} \begin{bmatrix} 0 & \frac{-2}{h(h+{h_{1}^{1}})}\\ 0 & 0 \end{bmatrix} \end{small}, \quad C_{2}= \begin{small} \begin{bmatrix} 0 & 0\\ \frac{-1}{h^{2}} & 0 \end{bmatrix} \end{small}. \end{array} $$

Proceeding as in Section 3.3 we find after some calculations discrete BJM parameters of the form (3.19), but with $E_{2}(\tilde {h})$ replaced by

$$ \tilde{E}_{2}(\tilde{h}^{1},\tilde{h}^{2})=\frac{2(\eta \tilde{h}^{1}\tilde{h}^{2}+2)(\tilde{h}^{1}+\tilde{h}^{2})}{D_{2}(\eta,h,\tilde{h}^{1},\tilde{h}^{2})} $$

with

$$ \begin{array}{@{}rcl@{}} D_{2}(\eta,h,\tilde{h}^{1},\tilde{h}^{2})&=& h^{3}\tilde{h}^{1}\tilde{h}^{2} (\tilde{h}^{1}+\tilde{h}^{2})(\tilde{h}^{1}+h)\eta^{2} + 2 h^{2}(\tilde{h}^{1}+\tilde{h}^{2})(h+\tilde{h}^{1})(h+\tilde{h}^{2})\eta\\ &&+4 h^{2}(h+\tilde{h}^{1}+\tilde{h}^{2}). \end{array} $$

**Fig. 6:**
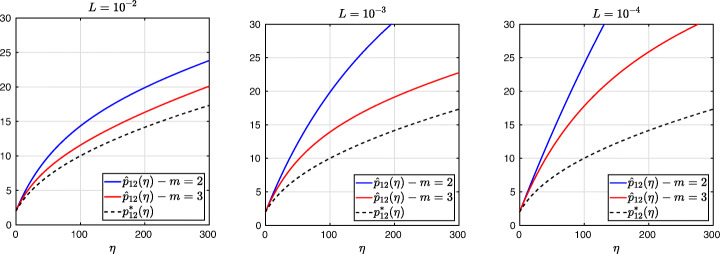
Comparison of the Robin parameters $p_{12}^{\ast }$ of the OSM and $\hat {p}_{12}$ of the BJM for *m* = 2,3,4 stretched (optimized) coarse points and overlap *L* = 10^− 2^ (left), *L* = 10^− 3^ (middle), *L* = 10^− 4^ (right)

**Fig. 7:**
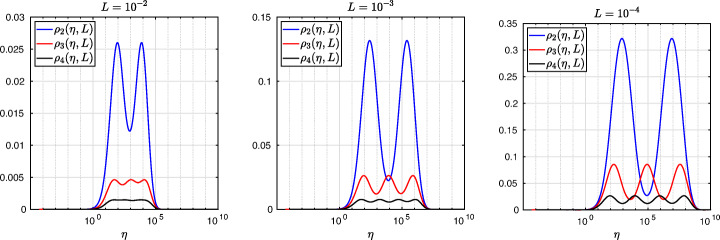
Convergence factors *ρ*_*m*_(*η*,*L*) as functions of *η* for *m* = 2,3,4 stretched (optimized) coarse points and *L* = 10^− 2^ (left), *L* = 10^− 3^ (middle), *L* = 10^− 4^ (right). Notice the different scales of the three plots

**Fig. 8:**
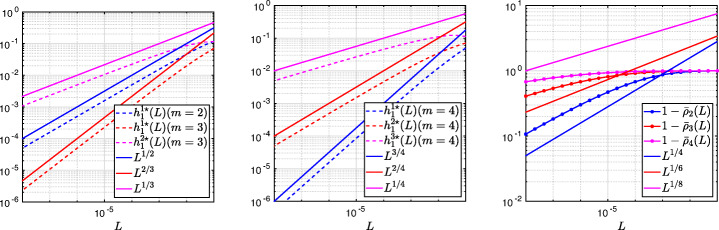
Left: *h*^*j*⋆^(*L*) versus *L* for *m* = 2,3. Middle: *h*^*j*⋆^(*L*) versus *L* for *m* = 4. Right: $1-\bar {\rho }_{m}(L)$ versus *L* for *m* = 2,3,4 stretched (optimized) coarse points

We now use the relations ${h_{1}^{2}}=1-\beta -{h_{1}^{1}}-h$ and ${h_{2}^{2}}=\alpha -{h_{2}^{1}}-h$, and take the limit for $h \rightarrow 0$ to get the continuous Robin parameters of the BJM (3.20) with the rational function $R_{2}(\tilde {L})$ replaced by

$$ \tilde{R}_{2}(\tilde{L},\tilde{h}^{1}) := \frac{\tilde{L}(\tilde{h}^{1})^{2}(\tilde{L}-\tilde{h}^{1})\eta^{2}+2\tilde{L}^{2}\eta+4}{2\tilde{L}\tilde{h}^{1}(\tilde{L}-\tilde{h}^{1})\eta+4\tilde{L}}, $$

which shows that the coefficients in the rational function in *η* can now be controlled by the mesh parameter $\tilde {h}^{1}$! To understand the impact of this new degree of freedom from the coarse mesh, we assume for simplicity that $\alpha =\frac {1-L}{2}$ and $\beta =\frac {1+L}{2}$, and ${h_{1}^{1}}={h_{2}^{1}}$ and ${h_{1}^{2}}={h_{2}^{2}}$. Inserting $\hat {p}_{12}$ and $\hat {p}_{21}$ into (3.5) and minimizing the maximum of the resulting convergence factor (3.5) over all frequencies *η* (using the MATLAB function fminunc), we find the best choice for the mesh stretching $h_{1}^{1\star }(L)$ that makes the convergence factor as small as possible. We show in Fig. 6 the behavior of the Robin parameter $\hat {p}_{12}(\eta )$ (blue lines) compared to the OSM parameter $p^{\star }_{12}(\eta )$ (black dashed lines) for different overlaps *L*. Clearly, the curves are very different from the ones corresponding to the uniform mesh (Fig. 2) which are very stable with respect to the overlap *L*. In the stretched case, the coarse mesh is strongly influenced by the overlap: the smaller the overlap, the more work needs/can be done in the optimization of the coarse points. The corresponding convergence factors are shown in Fig. 7 (blue lines), where one can now observe how they have two maxima. Hence, the optimization of the coarse points is solved when an equioscillation of the two maxima is obtained. If one compares these plots to the ones presented in Fig. 3, the enormous improvement obtained by optimizing the position of the *m* = 2 coarse points is clearly visible. This behavior is even more evident if one compares the deterioration of $\bar {\rho }_{2}$ of Fig. 4 (right) with the corresponding one of Fig. 8 (right - blue line): we observe that now the deterioration of the contraction factors with respect of the overlap is $\bar {\rho }_{2}(L)=1-O(L^{\frac {1}{4}})$. In Fig. 8 (left - blue line) we show the dependence of the optimized mesh position $h_{1}^{1\star }$ on *L*. We observe that

$$ h_{1}^{1\star}=O(L^{\frac{1}{2}}) \quad \text{for $m=2$}. $$

**Fig. 9:**
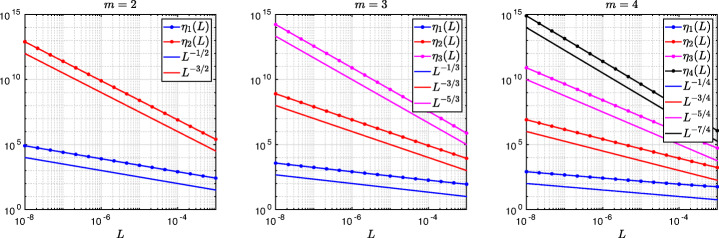
Maximum points *η*_*j*_(*L*) for *m* = 2 (left), *m* = 3 (middle) and *m* = 4 (right)

Finally, in Fig. 9 (left) we show the dependence of the frequencies *η*_1_ and *η*_2_ (the maximum points) on *L* and we observe that

$$ \eta_{1}=O(L^{-\frac{1}{2}}), \quad \eta_{2}=O(L^{-\frac{3}{2}}) \quad \text{for $m=2$}. $$

We prove these numerical observations in the next theorem.

#### **Theorem 3.6**

(Optimized stretched grid for *m* = 2) The Bank–Jimack Algorithm 1 with partition of unity (2.8), overlap *L*, and two equal subdomains $\alpha =\frac {1-L}{2}$ and $\beta =\frac {1+L}{2}$ has for *m* = 2 and overlap *L* small the optimized stretched grid points and associated contraction factor

3.21 $$ h_{1}^{1\star}=h_{2}^{1\star}=\frac{1}{2}\sqrt{L},\quad \bar{\rho}_{2}(L)=1-8\sqrt{2}L^{\frac14}+O(\sqrt{L}). $$

#### *Proof*

The system of equations satisfied when the maxima of *ρ*_2_(*η*,*L*) equioscillate as shown at the optimum in Fig. 3 is

3.22 $$ \rho_{2}(\eta_{1},L)=\rho_{2}(\eta_{2},L),\quad \partial_{\eta}\rho_{2}(\eta_{1},L)=0,\quad \partial_{\eta}\rho_{2}(\eta_{2},L)=0. $$

To solve this non-linear system asymptotically, we insert the ansatz $h_{1}^{1\star }=h_{2}^{1\star }:=C_{h^{1}}\sqrt {L}$ and $\eta _{1}:=C_{\eta _{1}}L^{-\frac 12}$ and $\eta _{2}:=C_{\eta _{2}}L^{-\frac 32}$ into the system (3.22), expand for overlap *L* small and find the relations

$$ \frac{2(C_{\eta_{1}}C_{h^{1}} + 4)}{\sqrt{C_{\eta_{1}}}}= \frac{C_{\eta_{2}}C_{h^{1}} + 4}{\sqrt{C_{\eta_{2}}}C_{h^{1}}}, \quad C_{\eta_{1}}C_{h^{1}}=4,\quad C_{\eta_{2}}C_{h^{1}}=4. $$

The solution is $ C_{\eta _{1}}= C_{\eta _{2}}=8$ and $C_{h^{1}}=\frac 12$, which leads when inserted with the ansatz into *ρ*_2_(*η*_1_,*L*) to (3.21) after a further expansion for *L* small. □

We thus conclude that the convergence factor of the BJM with an optimized stretched coarse mesh with *m* = 2 points behaves better than the convergence factor of the OSM with Robin transmission conditions which is $\rho _{OSM}= 1-O(L^{\frac {1}{3}})$, but worse than OSM with second order (Ventcell) transmission conditions, which is $\rho _{OSM}= 1-O(L^{\frac {1}{5}})$; see [14].

Let us now consider the case of *m* = 3 non-uniformly distributed coarse points with sizes ${h_{1}^{1}}$, ${h_{1}^{2}}$, and ${h_{1}^{3}}$, see Fig. 5 (fourth and fifth rows). Notice also the geometric relations ${h_{2}^{3}}=\alpha -(h+{h_{2}^{1}}+{h_{2}^{2}})$ and ${h_{1}^{3}}=1-\beta -(h+{h_{1}^{1}}+{h_{1}^{2}})$. Similar calculations as before (see also [21]) lead after expanding for *h* going to zero to the continuous Robin parameters of the BJM (3.20) with the rational function $R_{2}(\tilde {L})$ replaced by

$$ \tilde{R}_{3}(\tilde{L},\tilde{h}^{1},\tilde{h}^{2}):=\textstyle \frac{(\tilde{h}^{1})^{2}\tilde{h}^{2}(\tilde{h}^{1} + \tilde{h}^{2})(\tilde{L} - \tilde{h}^{1})(\tilde{L} - \tilde{h}^{1} - \tilde{h}^{2})\eta^{3} + 2(\tilde{h}^{1} + \tilde{h}^{2})(\tilde{L} - \tilde{h}^{1})(\tilde{L}\tilde{h}^{1} + \tilde{L}\tilde{h}^{2} - 2\tilde{h}^{1}\tilde{h}^{2} - (\tilde{h}^{2})^{2})\eta^{2} + 4\tilde{L}^{2}\eta+8}{2\tilde{h}^{1}\tilde{h}^{2}(\tilde{h}^{1} + \tilde{h}^{2})(\tilde{L} - \tilde{h}^{1})(\tilde{L} - \tilde{h}^{1} - \tilde{h}^{2})\eta^{2} + 4(\tilde{h}^{1} + \tilde{h}^{2})(\tilde{L} - \tilde{h}^{2})(\tilde{L} - \tilde{h}^{1})\eta + 8\tilde{L}}. $$

We thus have now two parameters from the stretched mesh from each side to optimize the convergence factor! We set again $\alpha =\frac {1-L}{2}$ and $\beta =\frac {1+L}{2}$, and ${h_{1}^{j}}={h_{2}^{j}}$, *j* = 1,2,3, and inserting $\hat {p}_{12}$ and $\hat {p}_{21}$ into the convergence factor (3.5) and minimizing the maximum of the resulting convergence factor over all frequencies *η*, we find the best choice for the mesh stretching $h_{1}^{1\star }(L)$, $h_{1}^{2\star }(L)$ that makes the convergence factor as small as possible, shown in Fig. 7 for a typical example in red. We notice that now three local maxima are present and equioscillate. In Fig. 8 (left), we show how the optimized choice of the stretched mesh parameters $h_{1}^{1\star }(L)$, $h_{1}^{2\star }(L)$ decay when the overlap *L* becomes small, and observe that

$$ h_{1}^{1\star}=O(L^{\frac{2}{3}}), \quad h_{1}^{2\star}=O(L^{\frac{1}{3}}) \quad \text{for $m=3$}. $$

Similarly, in Fig. 9 (middle) we find for the maximum points *η*_1_, *η*_2_, and *η*_3_ the asymptotic behavior

$$ \eta_{1}=O(L^{-\frac{1}{3}}), \quad \eta_{2}=O(L^{-\frac{3}{3}}), \quad \eta_{3}=O(L^{-\frac{5}{3}}) \quad \text{for $m=3$}. $$

#### **Theorem 3.7**

(Optimized stretched grid for *m* = 3) Under the same assumptions as in Theorem 3.6, the Bank–Jimack Algorithm 1 has for *m* = 3 and overlap *L* small the optimized stretched grid points and associated contraction factor

3.23 $$ h_{1}^{1\star}=h_{2}^{1\star}=\frac12L^{\frac23},\quad h_{1}^{2\star}=h_{2}^{2\star}=\frac12L^{\frac13},\quad \bar{\rho}_{3}(L)=1-8\sqrt{2}L^{\frac16}+O(L^{\frac13}). $$

#### *Proof*

The system of equations satisfied when the maxima of *ρ*_2_(*η*,*L*) equioscillate as shown at the optimum in Fig. 3 is

3.24 $$ \begin{array}{ll} &\rho_{2}(\eta_{1},L)=\rho_{2}(\eta_{2},L),\quad \rho_{2}(\eta_{2},L)=\rho_{2}(\eta_{3},L),\\ &\partial_{\eta}\rho_{2}(\eta_{1},L)=0,\quad \partial_{\eta}\rho_{2}(\eta_{2},L)=0,\quad \partial_{\eta}\rho_{2}(\eta_{3},L)=0. \end{array} $$

Inserting the ansatz $h_{1}^{1\star }=h_{2}^{1\star }:=C_{h^{1}}L^{\frac 23}$, $h_{1}^{2\star }=h_{2}^{2\star }:=C_{h^{2}}L^{\frac 13}$, and $\eta _{1}:=C_{\eta _{1}}L^{-\frac 13}$, $\eta _{2}:=C_{\eta _{2}}L^{-\frac 33}$, $\eta _{2}:=C_{\eta _{2}}L^{-\frac 53}$ into the system (3.24), we can solve the system asymptotically for the constants when the overlap *L* becomes small, which leads to (3.23). □

The analysis for *m* = 4 stretched coarse points follows the same lines, and we find after a longer computation for the continuous Robin parameters of the BJM (3.20) with the rational function $R_{2}(\tilde {L})$ replaced by (see also [21] for details)

$$ \tilde{R}_{4}(\tilde{L},\tilde{h}^{1},\tilde{h}^{2},\tilde{h}^{3}) = \frac{\tilde{N}_{4}(\tilde{L},\tilde{h}^{1},\tilde{h}^{2},\tilde{h}^{3})} {\tilde{D}_{4}(\tilde{L},\tilde{h}^{1},\tilde{h}^{2},\tilde{h}^{3})} $$

with the numerator and denominator given by

$$ \begin{array}{@{}rcl@{}} \tilde{N}_{4}&=& (\tilde{h}^{1})^{2}\tilde{h}^{2}\tilde{h}^{3}(\tilde{h}^{3} + \tilde{h}^{2})(\tilde{h}^{2} + \tilde{h}^{1})(\tilde{L} - \tilde{h}^{1} - \tilde{h}^{2})(\tilde{L} - \tilde{h}^{1} - \tilde{h}^{2} - \tilde{h}^{3})\eta^{4} \\ &&+ 2(\tilde{L} - \tilde{h}^{1} - \tilde{h}^{2})(\tilde{h}^{3} + \tilde{h}^{2})(\tilde{h}^{2} + \tilde{h}^{1})\big((\tilde{L} - 2\tilde{h}^{3})(\tilde{h}^{1})^{2}-(\tilde{h}^{1})^{3}\\ && + \tilde{h}^{3}(\tilde{L} - 2\tilde{h}^{2} - \tilde{h}^{3})\tilde{h}^{1} + \tilde{h}^{3}\tilde{h}^{2}(\tilde{L} - \tilde{h}^{2} - \tilde{h}^{3})\big)\eta^{3} + \big((8\tilde{h}^{2} + 8\tilde{h}^{3}-4\tilde{L})(\tilde{h}^{1})^{3}\\ && + 4(\tilde{L} - \tilde{h}^{2} - \tilde{h}^{3})(\tilde{L} - 3\tilde{h}^{2} - 3\tilde{h}^{3})(\tilde{h}^{1})^{2} + 8(\tilde{h}^{3} + \tilde{h}^{2})((\tilde{h}^{2})^{2}/2 + ((5\tilde{h}^{3})/2-2\tilde{L})\tilde{h}^{2} \\ && + \tilde{L}^{2} - 2\tilde{L}\tilde{h}^{3} + (\tilde{h}^{3})^{2}/2)\tilde{h}^{1} + 4((\tilde{L} - 2\tilde{h}^{3})\tilde{h}^{2} + \tilde{h}^{3}(\tilde{L} - \tilde{h}^{3}))(\tilde{h}^{3} + \tilde{h}^{2})(\tilde{L} - \tilde{h}^{2})\big)\eta^{2}\\ && + 8\tilde{L}^{2}\eta+16,\\ \tilde{D}_{4}&=&2\tilde{h}^{1}\tilde{h}^{2}\tilde{h}^{3}(\tilde{h}^{3} + \tilde{h}^{2})(\tilde{h}^{2} + \tilde{h}^{1})(\tilde{L} - \tilde{h}^{1} - \tilde{h}^{2})(\tilde{L} - \tilde{h}^{1} - \tilde{h}^{2} - \tilde{h}^{3})\eta^{3}\\ &&+ 4(\tilde{L} - \tilde{h}^{1} - \tilde{h}^{2})(\tilde{h}^{3} + \tilde{h}^{2})(\tilde{h}^{2} + \tilde{h}^{1})\big((\tilde{L} - 2\tilde{h}^{3})\tilde{h}^{1} -(\tilde{h}^{1})^{2}+ \tilde{h}^{3}(\tilde{L} - \tilde{h}^{2} - \tilde{h}^{3})\big)\eta^{2}\\ &&+ \big((8\tilde{h}^{2} + 8\tilde{h}^{3}-8\tilde{L})(\tilde{h}^{1})^{2} + 8(\tilde{L} - \tilde{h}^{2} - \tilde{h}^{3})^{2}\tilde{h}^{1}\\ && + 8(\tilde{h}^{3} + \tilde{h}^{2})(\tilde{L} - \tilde{h}^{3})(\tilde{L} - \tilde{h}^{2})\big)\eta + 16\tilde{L}, \end{array} $$

which leads to the results shown in Figs. 7, 8 (middle), 9 (right), which show that

$$ h_{1}^{1\star}=O(L^{\frac{3}{4}}), \quad h_{1}^{2\star}=O(L^{\frac{2}{4}}), \quad h_{1}^{3\star}=O(L^{\frac{1}{4}}) \quad \text{for $m=4$}, $$

and for the maximum points we find

3.25 $$ \eta_{1}=O(L^{-\frac{1}{4}}), \quad \eta_{2}=O(L^{-\frac{3}{4}}), \quad \eta_{3}=O(L^{-\frac{5}{4}}), \quad \eta_{4}=O(L^{-\frac{7}{4}}) \quad \text{for $m=4$}. $$

#### **Theorem 3.8**

(Optimized stretched grid for *m* = 4) Under the same assumptions of Theorem 3.6, the Bank–Jimack Algorithm 1 has for *m* = 4 and overlap *L* small the optimized stretched grid points and associated contraction factor

$$ h_{1}^{1\star}=h_{2}^{1\star}=\frac12L^{\frac34},~ h_{1}^{2\star}=h_{2}^{2\star}=\frac12 L^{\frac24},~ h_{1}^{3\star}=h_{2}^{3\star}=\frac12 L^{\frac14},~ \bar{\rho}_{4}(L)=1-8\sqrt2L^{\frac18}+O(L^{\frac14}). $$

#### *Proof*

We proceed as in the proof of Theorems 3.6 and 3.7. □

These results for optimized stretched coarse grids with *m* = 2, *m* = 3, and *m* = 4 points lead us to formulate the following conjecture:

#### *Conjecture 3.9*

The Bank–Jimack Algorithm 1 with partition of unity (2.8), overlap *L*, and two equal subdomains $\alpha =\frac {1-L}{2}$ and $\beta =\frac {1+L}{2}$, has for overlap *L* small the optimized stretched grid point locations and associated contraction factor

$$ h_{1}^{j\star}=h_{2}^{j\star}\sim \frac12 L^{\frac{m-j}{m}},~~ j=1,2,\ldots,m-1,\quad \bar{\rho}_{m}(L) \sim 1-8\sqrt2L^{\frac{1}{2m}}. $$

This result shows that one should choose a geometric coarsening related to the overlap to form the outer coarse grid leading to the best performance for the Bank–Jimack domain decomposition algorithm. A practical approach is to just take a geometrically stretched grid with respect to the overlap size,

$$ h_{j}:=L^{\frac{m-1}{m}},\quad j=1,\ldots,m, $$

and then to sum the step sizes *h*_*j*_ and scale the result to the size of the outer remaining domain, say $\hat {L}$, to get the actual mesh sizes $\tilde {h}_{j}$ to use,

3.26 $$ s:=\sum\limits_{j=1}^{m}h_{j}=\frac{1-L}{1-L^{\frac{1}{m}}}\quad\Longrightarrow\quad \tilde{h}_{j}:=\frac{h_{j}}{s}\hat{L}=\frac{L^{-\frac{j}{m}}-L^{\frac{1-j}{m}}}{L^{-1}-1}\hat{L}. $$

**Fig. 10:**
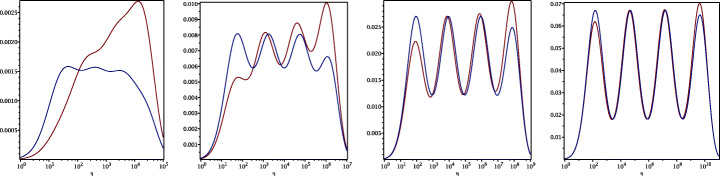
Asymptotic stretching from Conjecture 3.9 (red) compared to the direct geometric stretching in (3.26) (blue) for overlap sizes $L=\frac {1}{10^{j}}$, *j* = 2,3,4,5

This direct geometric stretching including the last grid cell is preasymptotically even a bit better, as one can see in Fig. 10.

## Numerical Experiments

In this section, we present numerical experiments to illustrate our theoretical results. We start with experiments for equally spaced coarse meshes, and compare their performance with the optimized geometrically stretched ones. We consider both a case of constant overlap *L* and a case where the overlap is proportional to the mesh size. We then also explore numerically the influence of coarsening the meshes in the direction tangential to the interface. In all these cases, we study the performance of the BJM as a stationary method and as a preconditioner for GMRES. We discretize the Poisson equation (2.5) (defined on a unit square *Ω* = (0,1)^2^) using *n*^2^ (interior) mesh points where *n* = 2^*ℓ*^ − 1, for *ℓ* = 5,6,7, is the number of interior points on the global fine mesh in each direction (Fig. 1). The results corresponding to a uniform coarsening in direction *x* are presented in Section 4.1. Section 4.2 focuses on optimized stretched coarsening in direction *x*. Finally, in Section 4.3 we study the effect of the coarsening in both directions *x* and *y*.

### Uniform Coarsening in Direction *x*

**Fig. 11:**
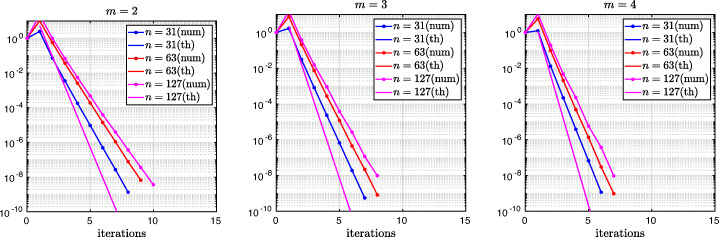
Decay of the error of the BJM (stationary) iteration for *m* = 2 (left), *m* = 3 (middle) and *m* = 4 (right) uniformly distributed coarse points (in direction *x*) and constant overlap $L=\frac {1}{16}$. Notice that, in each plot, the solid curves representing the theoretical convergence estimates coincide since they correspond to the same overlap *L*

**Fig. 12:**
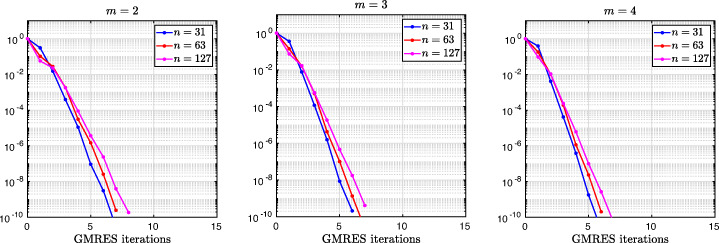
Decay of the residual of the GMRES iteration preconditioned by BJM for *m* = 2 (left), *m* = 3 (middle) and *m* = 4 (right) uniformly distributed coarse points (in direction *x*) and constant overlap $L=\frac {1}{16}$

We start with the equally spaced coarse mesh case, coarsened only along the *x* axis. At first, we consider the case with a constant overlap $L=\frac {1}{16}$, which corresponds to *n*_*s*_ = 3,5,9 for *ℓ* = 5,6,7, respectively. Moreover, to test the methods in the cases studied by our theoretical analysis, we consider *m* = 2,3,4 coarse mesh points. The results of the numerical experiments are shown in Figs. 11 and 12. The former shows the decay of the error corresponding to the BJM as a stationary iteration, while the latter presents the decay of the GMRES residuals along the iterations. All the plots show that the effect of the number of coarse points on the convergence is very mild. This corresponds to the results discussed in Section 3.3 and shown in Fig. 4 (right): if the overlap *L* is constant, the contraction factor does not improve significantly if more (uniformly distributed) coarse points are considered. The same effect can be observed in the GMRES convergence.

**Fig. 13:**
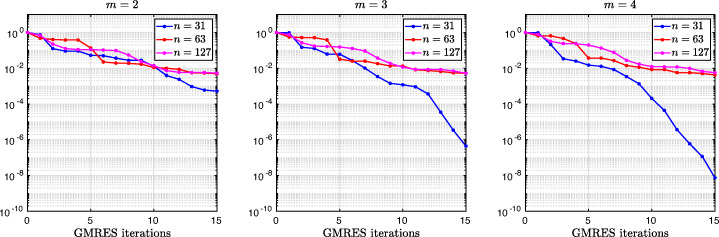
Decay of the residual of the GMRES iteration preconditioned by BJM with the original partition of unity used in [3] for *m* = 2 (left), *m* = 3 (middle) and *m* = 4 (right) uniformly distributed coarse points (in direction *x*) and constant overlap $L=\frac {1}{16}$

Now, we wish to study the effect of the new partition of unity proposed in [10] and constructed using (2.3). This was used in all the experiments discussed above. If we use the original partition of unity, we already know from [10] that the BJM does not converge as a stationary method. Therefore, we use it only as a preconditioner for GMRES and obtain the results depicted in Fig. 13. By comparing the results of this figure with the ones of Fig. 12, we see that the effect of the new partition of unity is tremendous: GMRES converges much faster and is very robust against mesh refinements. For further information on the influence of the partition of unity on Schwarz methods, see [16].

**Fig. 14:**
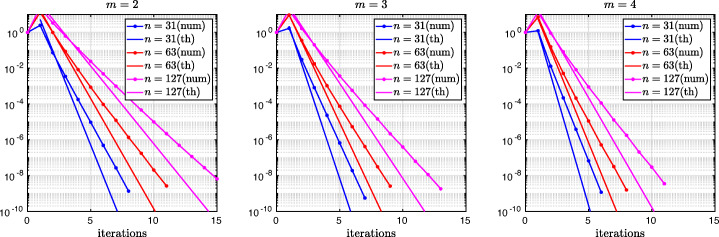
Decay of the error of the BJM (stationary) iteration for *m* = 2 (left), *m* = 3 (middle) and *m* = 4 (right) uniformly distributed coarse points (in direction *x*) and overlap *L* = 2*h*

**Fig. 15:**
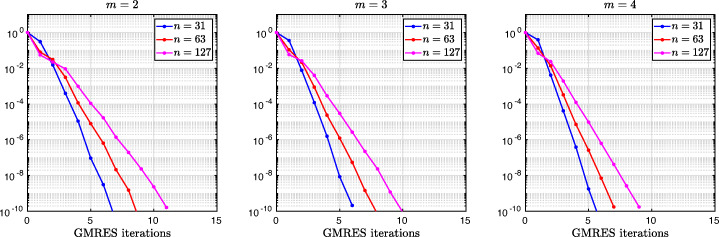
Decay of the residual of the GMRES iteration preconditioned by BJM and for *m* = 2 (left), *m* = 3 (middle) and *m* = 4 (right) uniformly distributed coarse points (in direction *x*) and overlap *L* = 2*h*

**Fig. 16:**
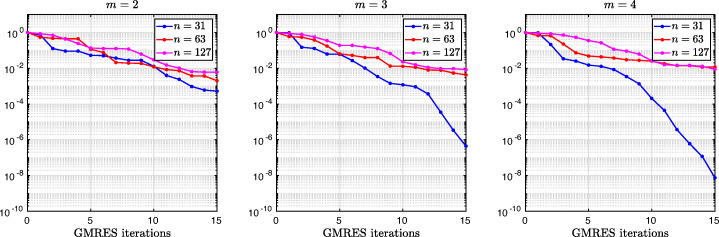
Decay of the residual of the GMRES iteration preconditioned by BJM with the original partition of unity used in [3] for *m* = 2 (left), *m* = 3 (middle) and *m* = 4 (right) uniformly distributed coarse points (in direction *x*) and overlap *L* = 2*h*

Now, let us now consider an overlap proportional to the mesh size, namely *L* = 2*h*, and repeat the experiments already described. The corresponding results are shown in Figs. 14, 15 and 16. As before, we observe that the BJM method (as stationary iteration and as preconditioner) is robust against the number of coarse mesh points. In this case, the convergence deteriorates with mesh refinement since the overlap *L* gets smaller proportionally to *h*. Finally, we observe again the great impact of the new partition of unity by comparing Figs. 15 and 16.

### Stretched Coarsening in Direction *x*

**Fig. 17:**
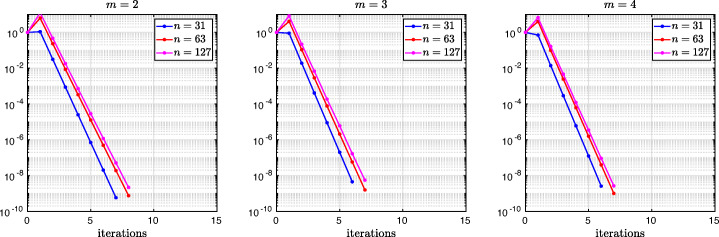
Decay of the error of the BJM (stationary) iteration for *m* = 2 (left), *m* = 3 (middle) and *m* = 4 (right) stretched (optimized) coarse points (in direction *x*) and constant overlap $L=\frac {1}{16}$

**Fig. 18:**
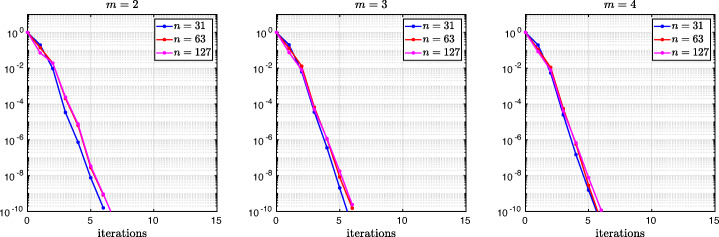
Decay of the residual of the GMRES iteration preconditioned by BJM and for *m* = 2 (left), *m* = 3 (middle) and *m* = 4 (right) stretched (optimized) coarse points (in direction *x*) and constant overlap $L=\frac {1}{16}$

**Fig. 19:**
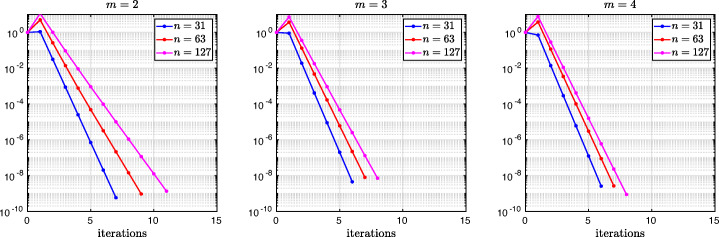
Decay of the error of the BJM (stationary) iteration for *m* = 2 (left), *m* = 3 (middle) and *m* = 4 (right) stretched (optimized) coarse points (in direction *x*) and overlap *L* = 2*h*

**Fig. 20:**
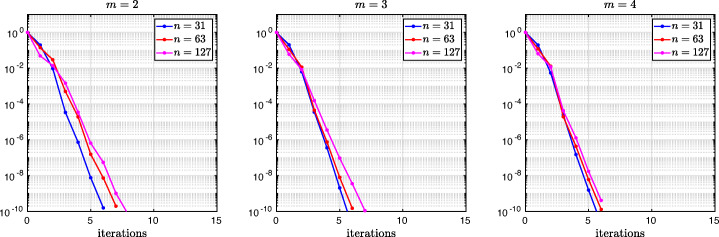
Decay of the residual of the GMRES iteration preconditioned by BJM for *m* = 2 (left), *m* = 3 (middle) and *m* = 4 (right) stretched (optimized) coarse points (in direction *x*) and overlap *L* = 2*h*

In this section, we repeat the experiments presented in Section 4.1, but we optimize the position of the coarse mesh points by minimizing numerically the contraction factor (as in Section 3.4). We begin with the case of constant overlap $L=\frac {1}{16}$. The corresponding numerical results are shown in Figs. 17 and 18. These results show that optimizing the coarse mesh leads to a faster method which is robust against the mesh refinement. However, due to the constant overlap, there is only little improvement with respect to the constant coarsening case. To better appreciate the effect of the mesh optimization, we consider the case with overlap *L* = 2*h*. The corresponding results are shown in Figs. 19 and 20. By comparing these results with the ones of Figs. 14 and 15, one can see clearly the improvement of the BJM convergence: the number of iterations (for both stationary and preconditioned GMRES methods) are essentially halved in the case of finer meshes.

### Coarsening in Direction *x* and *y*

**Fig. 21:**
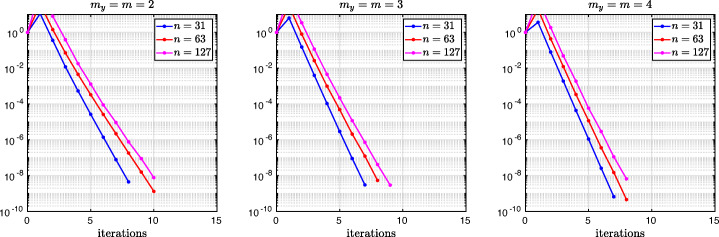
Decay of the error of the BJM (stationary) iteration for *m*_*y*_ = *m* = 2 (left), *m*_*y*_ = *m* = 3 (middle) and *m*_*y*_ = *m* = 4 (right) uniformly distributed coarse points (in direction *x* and *y*) and constant overlap $L=\frac {1}{16}$

**Fig. 22:**
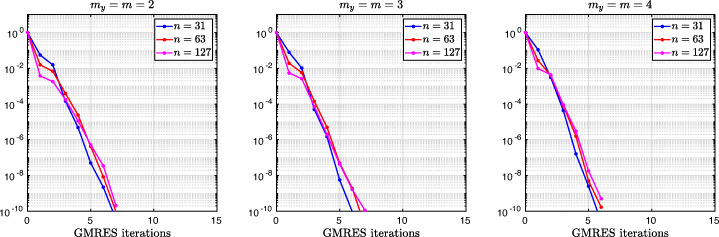
Decay of the residual of the GMRES iteration preconditioned by BJM for *m*_*y*_ = *m* = 2 (left), *m*_*y*_ = *m* = 3 (middle) and *m*_*y*_ = *m* = 4 (right) uniformly distributed coarse points (in direction *x* and *y*) and constant overlap $L=\frac {1}{16}$

**Fig. 23:**
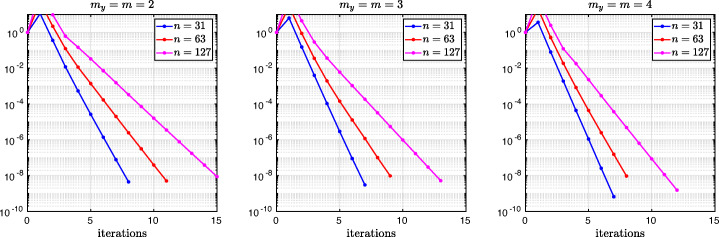
Decay of the error of the BJM (stationary) iteration for *m*_*y*_ = *m* = 2 (left), *m*_*y*_ = *m* = 3 (middle) and *m*_*y*_ = *m* = 4 (right) uniformly distributed coarse points (in direction *x* and *y*) and overlap *L* = 2*h*

**Fig. 24:**
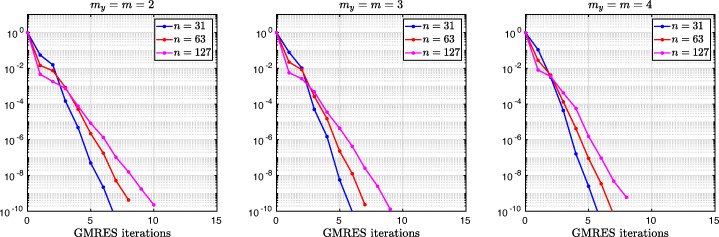
Decay of the residual of the GMRES iteration preconditioned by BJM for *m*_*y*_ = *m* = 2 (left), *m*_*y*_ = *m* = 3 (middle) and *m*_*y*_ = *m* = 4 (right) uniformly distributed coarse points (in direction *x* and *y*) and overlap *L* = 2*h*

We conclude our numerical experiments by studying the effect of a (uniform) coarsening in both *x* and *y* directions. As before, we consider both cases $L=\frac {1}{16}$ and *L* = 2*h*. The results shown in Figs. 21, 22, 23 and 24 indicate that a coarsening in direction *y* does not have a significant impact on the convergence of the BJM method.

## Conclusions

We provided a detailed convergence analysis of the Bank–Jimack domain decomposition method for the Laplace problem and two subdomains. Our analysis reveals that one should coarsen the outer mesh each subdomain uses in a geometric progression related to the size of the overlap if one wants to get good convergence, and arbitrarily weak dependence on the overlap size is possible (see also [17] for a different technique reaching this). In order for these results to hold one has to use a slightly modified partition of unity in the Bank–Jimack algorithm, without which the convergence of the method is much worse. We obtained our results by an asymptotic process as the subdomain mesh size goes to zero, and thus the results hold at the continuous level.

A possibility for further optimization at the discrete level is the observation that the maxima in the optimized method, shown in Fig. 7, occur for very high values of *η* which represent a Fourier frequency, and thus may lie outside of the frequencies representable on the mesh used. This can be seen quantitatively for example from the stretched case for *m* = 4, where the largest $\eta _{4}=O(L^{-\frac 74})$, and the highest Fourier frequency can be estimated as *η* = *O*(*h*^− 1^), see [14]. Hence, if the overlap is of the order of the mesh size, *L* = *h*, *η*_4_ would be already much larger than what the grid can represent, and we see in fact from (3.25) that only half the number of bumps would need to be taken in consideration for the optimization.

## Footnotes

1 BJM is also defined for non-self-adjoint problems. We assume this here only to simplify the notation.

2 This problem does not appear in the formulation based on Lagrange multipliers proposed in [5].

3 In our numerical experiments, we will test also coarsening in both directions.

4 Notice that the first coarse points, namely the point number *n*_1_ + *n*_*s*_ + 1 for the first mesh and the point number *m*_1_ for the second mesh, are located at distance *h* from the interfaces. This choice is motivated by the fact that we will define discrete (finite-difference) derivatives across these points and then in Section 3 take limits for $h\rightarrow 0$, while keeping the numbers *m*_1_ and *m*_2_ of coarse points fixed.

5 The 1 in the partitions of unity *D*_1_ and *D*_2_ is however very important, see [10], and for more details on whether partition of unity functions influence the convergence of Schwarz methods, see [16].

6 Notice that similar results are proved in [4] and [1] using Tchebyshev polynomials and three-term recurrence relations.

7 To evaluate the convergence factor numerically one needs to factor out an exponential in the hyperbolic trigonometric functions to avoid overflow.

8 Note that one of these grid points was merged into the interface when taking the limit as *h* goes to zero, so the grid has *m* = 2 mesh cells of the same size, with only *m* − 1 = 1 grid point in the middle left. The same also happens for other values of *m*, there are *m* mesh cells, but only *m* − 1 grid points separating them in the outer grid.
